# Transcriptional transitions in Alphonso mango (*Mangifera indica* L.) during fruit development and ripening explain its distinct aroma and shelf life characteristics

**DOI:** 10.1038/s41598-017-08499-5

**Published:** 2017-08-18

**Authors:** Ashish B. Deshpande, Krishanpal Anamika, Vineet Jha, Hemangi G. Chidley, Pranjali S. Oak, Narendra Y. Kadoo, Keshav H. Pujari, Ashok P. Giri, Vidya S. Gupta

**Affiliations:** 1 Plant Molecular Biology Group, Biochemical Sciences Division, CSIR-National Chemical Laboratory, Pune, 411008 Maharashtra India; 2 Labs, Persistent Systems Limited, Pingala-Aryabhata, Erandwane, Pune, 411004 India; 3 Dr. Balasaheb Sawant Konkan Agriculture University, Dapoli, 415712 Maharashtra India

## Abstract

Alphonso is known as the “King of mangos” due to its unique flavor, attractive color, low fiber pulp and long shelf life. We analyzed the transcriptome of Alphonso mango through Illumina sequencing from seven stages of fruit development and ripening as well as flower. Total transcriptome data from these stages ranged between 65 and 143 Mb. Importantly, 20,755 unique transcripts were annotated and 4,611 were assigned enzyme commission numbers, which encoded 142 biological pathways. These included ethylene and flavor related secondary metabolite biosynthesis pathways, as well as those involved in metabolism of starch, sucrose, amino acids and fatty acids. Differential regulation (p-value ≤ 0.05) of thousands of transcripts was evident in various stages of fruit development and ripening. Novel transcripts for biosynthesis of mono-terpenes, sesqui-terpenes, di-terpenes, lactones and furanones involved in flavor formation were identified. Large number of transcripts encoding cell wall modifying enzymes was found to be steady in their expression, while few were differentially regulated through these stages. Novel 79 transcripts of inhibitors of cell wall modifying enzymes were simultaneously detected throughout Alphonso fruit development and ripening, suggesting controlled activity of these enzymes involved in fruit softening.

## Introduction

Mango (*Mangifera indica* L.) is one of the most popular and highly favored fruit. Global mango production was reported to be 43.3 million metric tons in 2013 preceding banana, apple, grape and orange. (https://www.statista.com/statistics/237064/top-world-producers-of-selected-fresh-fruit-by-value-2009/). There are thousands of mango cultivars worldwide, among which Alphonso, Keitt, Kent, Lilli, Zill, Osteen, Haden, Kesar, Pairi, Dashehari, Langra and Banganapalli are well known. These varieties vary in their fruit color, size, shape, flavor, taste and ripening period and pattern. To understand the composition and biosynthesis of their unique flavor and complex ripening process various studies have been carried out at metabolic^1–3^, proteomic^4–6^, genetic^7–15^ and post-harvest processing^16–20^ levels. Whole genome sequencing and RNA sequencing (RNAseq) are the two important high throughput technologies recently adopted to understand the complex cellular and physiological processes in fruits such as citrus^21^, tomato^22^ and strawberry^23^ as well as domestication and diseases tolerance in citrus^21, 24^. Although mango genome sequence is yet not available, few recent studies have described the transcriptomic analysis from various tissues of few mango cultivars. The first report from Zill mango^25^ provided extensive transcriptomic and proteomic profiling from pulp and skin tissues of four fruit developing stages using pooled RNA but not stage specific and differentially expressed transcripts. Another study of leaf transcriptome and chloroplast genome sequencing from cultivar Langra provided information about the production of several bioactive compounds^26^. Transcriptome analysis from two (raw and ripe) and three (raw, mid ripe and ripe) stages of Kent^27^ and Dashehari fruit pulp^28^, respectively provided important insights into the ripening process and flavor biogenesis in these mango cultivars.

India is the largest producer and exporter of mango with 40.6% share in international mango market (http://www.fao.org). Among the Indian mango cultivars Alphonso is globally favored and highly exported mango due to its unique and attractive flavor, low fiber containing pulp and high carotene content^29, 30^. The ripening duration of Alphonso mango is 15 days from harvest, which is the highest among all mango cultivars; for example, the ripening duration for Kent and Dashehari mango fruit is 10 and 6 days, respectively^27, 28^. Fruit ripening in Alphonso mango progresses from skin towards the stone leading to attractive skin color and easy monitoring of ripening progress. On the other hand various mango varieties, *viz*. Haden, Keitt, Kent, Tommy Atkins (National Mango Board, USA; http://www.mango.org) and Dashehari^28^ show polarity of their ripening from fruit stone to skin, making it difficult to identify ripened fruits. Longer ripening duration and shelf life of Alphonso mango provides sufficient time for its transportation globally. The mechanisms underlying these unique properties of Alphonso mango need to be explored in depth at spatial and temporal level of fruit development and ripening using transcriptomic, proteomic and metabolomic approaches as they can be correlated to the specific phenotype. In the present study, we performed transcriptome analysis of Alphonso mango through eight different tissues such as flower, whole fruit at 30 and 60 DAP (Days After Pollination), pulp and skin of 90 DAP fruit (mature raw fruit) and pulp from three fruit ripening stages i.e. 5 DAH (Days After Harvest): table green stage; 10 DAH: mid ripe stage and 15 DAH: ripe stage to analyze various fruit developing and ripening processes in Alphonso mango.

## Results

### Alphonso mango transcriptome

Alphonso mango transcriptome was screened through eight tissue samples. To map differentially expressed transcripts a merged assembly was generated from the reads of all the tissues, which reflected upon overall Alphonso mango flower and fruit transcriptome. For each tissue read numbers were more than 100 million, which were assembled using k-mers 67, 75 and 83 separately and then merged for individual tissue. Average number of unique transcripts post assembly was 76,043 and with N50 and N80 values as 1,835 and 1,008 bp, respectively (Table 1). The minimum and maximum lengths of transcript from these assemblies were 100 and 17,342 bp, respectively (Table 1). Average number of transcripts was 11,925 upon filtering for redundancy and identifying candidate coding regions with maximum 90% identity and minimum 70% coverage (Table 2).

**Table 1:** Alphonso *de novo* transcriptome assembly statistics.

Library	k-mer	Number of transcripts	Total Loci	Max transcript length (bp)	Min transcript length (bp)	Average transcript length (bp)	Total assembled transcript length (bp)	N (ambiguous base) in Assembly	% of N in Assembly	Transcript >100 bases	Transcript >500 bases	Transcript >1000 bases	Transcript >10000 bases	N50	N80
Flower	67	93034	52657	11812	67	1258.42	117076274	19738	0.000168591	92734	65039	46602	11	1878	1038
Flower	75	77624	49072	9554	75	1190.5	92411541	8636	9.35E-05	77529	53307	36738	0	1767	948
Flower	83	59964	43216	8515	83	1088.82	65289867	1718	2.63E-05	59950	39858	25880	0	1607	819
Flower	Merge	108468	25968	11810	100	1316.51	142798983	0	0	108459	86918	57729	7	1792	991
30DAP	67	86382	47302	15826	67	1323.05	114287275	35912	0.000314226	85831	60199	43912	78	2014	1101
30DAP	75	72205	44634	15531	75	1258.01	90834866	12431	0.000136853	72066	49617	34935	41	1896	1008
30DAP	83	56044	39936	12729	83	1142.61	64036591	3219	5.03E-05	56023	37033	24431	6	1720	858
30DAP	Merge	101839	22937	15923	100	1379.66	140503210	0	0	101831	82127	55153	51	1895	1032
60DAP	67	83471	45979	15420	67	1302.75	108741771	34825	0.000320254	82922	57930	41937	49	1980	1085
60DAP	75	68994	42677	15586	75	1251.44	86342100	13325	0.000154328	68845	47437	33343	19	1885	1002
60DAP	83	53149	37771	12269	83	1149.93	61117755	3912	6.40E-05	53112	35635	23635	2	1710	871
60DAP	Merge	97639	22857	15586	100	1367.7	133540400	0	0	97625	78534	52880	31	1879	1030
90DAP Skin	67	71479	41296	15966	67	1315.74	94048042	21996	0.00023388	71136	49940	36213	58	1990	1087
90DAP Skin	75	60601	39188	15506	75	1239.73	75128774	11071	0.00014736	60476	41350	28762	30	1874	985
90DAP Skin	83	47769	35414	14057	83	1114.63	53244933	3903	7.33E-05	47734	31184	20097	6	1683	820
90DAP Skin	Merge	83832	20646	17342	100	1360.29	114035452	0	0	83817	67358	44798	47	1866	1014
90DAP Pulp	67	44496	30174	15542	67	1290.02	57400897	4191	7.30E-05	44430	31603	22541	31	1903	1047
90DAP Pulp	75	40194	29670	13653	75	1175.86	47262652	2419	5.12E-05	40166	27424	18405	19	1741	911
90DAP Pulp	83	35021	28601	10602	83	1004.51	35179037	628	1.79E-05	35015	21568	13015	3	1530	704
90DAP Pulp	Merge	50448	15808	15542	100	1297.29	65445727	0	0	50445	40882	25935	28	1742	945
5DAH	67	53017	34113	14231	67	1243.82	65943359	6471	9.81E-05	52913	36300	25920	25	1882	1026
5DAH	75	44560	31464	12211	75	1188.26	52948984	1539	2.91E-05	44550	30232	20738	15	1775	939
5DAH	83	36283	28248	10605	97	1076	39040480	285	7.30E-06	36282	23661	15187	1	1595	798
5DAH	Merge	58650	16942	14390	100	1306.91	76650373	0	0	58645	47088	30652	26	1769	969
10DAH	67	49255	29960	12544	67	1335.9	65799836	7462	0.000113405	49143	35439	26041	7	1981	1112
10DAH	75	42952	28652	9554	75	1259.87	54114011	4163	7.69E-05	42910	30351	21523	0	1863	1016
10DAH	83	35672	26433	8215	83	1137.45	40575250	1056	2.60E-05	35665	24150	16127	0	1677	875
10DAH	Merge	55433	15560	14694	100	1406.1	77944611	0	0	55431	45727	31224	10	1901	1060
15DAH	67	46057	28952	10959	67	1294.4	59616054	5774	9.69E-05	45972	32815	23684	2	1913	1067
15DAH	75	40136	27796	9458	75	1214.11	48729396	3030	6.22E-05	40104	27750	19219	0	1805	962
15DAH	83	33362	25674	8195	86	1073.14	35801945	498	1.39E-05	33357	21850	13938	0	1598	795
15DAH	Merge	52033	14787	10959	100	1369.58	71263347	0.00E + 00	0	52029	43001	28703	3	1838	1023
All	Merge	434366	31629	15967	100	1326.31	576104058	0	0	434313	345481	224075	170	1835	972

**Table 2:** Number of non redundant (NR) transcripts/sample.

Assembly	No of NR transcripts
Flower	15778
30 DAP	14622
60 DAP	14090
90 DAP pulp	9382
90 DAP skin	12664
5 DAH	10084
10 DAH	9748
15 DAH	9032

**Figure 1:**
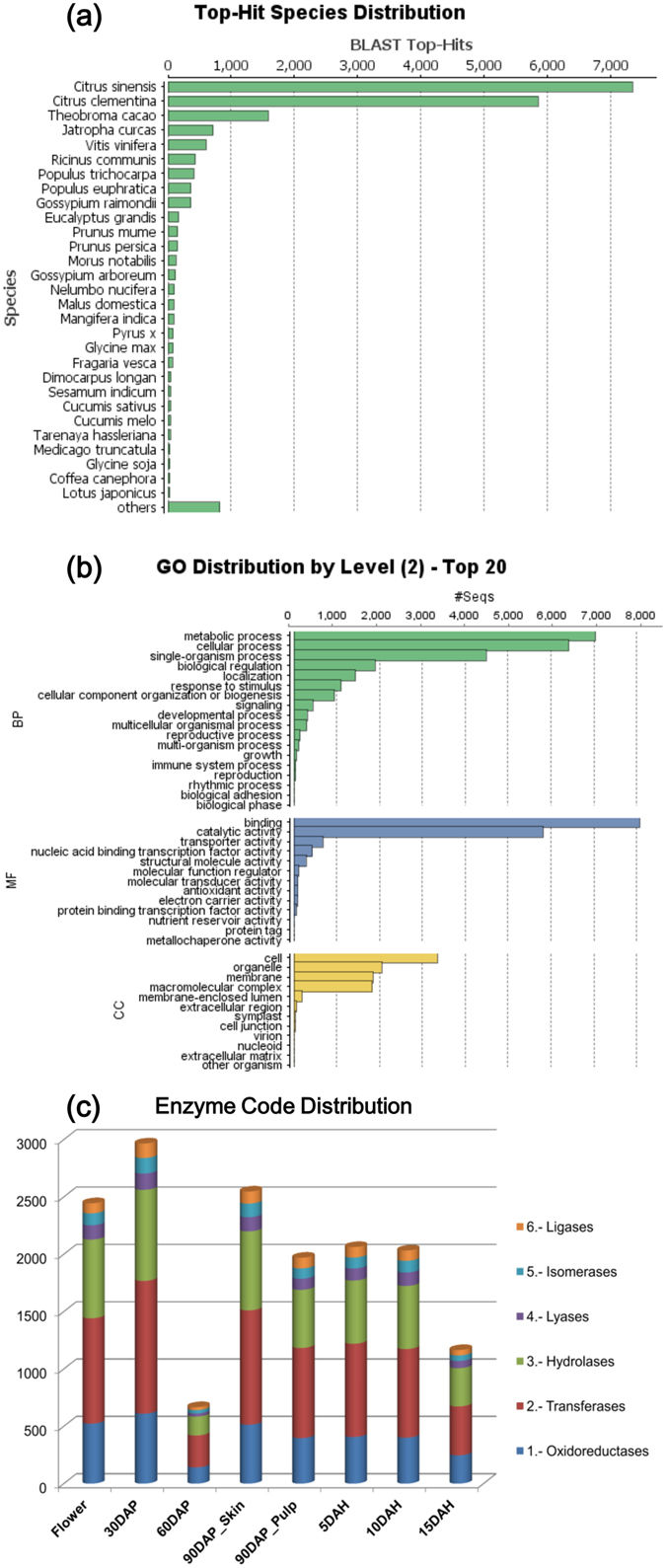
Blast statistics showing (**a**) distribution of top hit species, (**b**) distribution of top gene ontologies from BP: biological processes, MF: molecular functions and CC: cellular components and (**c**) Enzyme code distribution: number of transcripts (Y-axis) encoding six classes of enzymes through various stages of Alphonso mango fruit development and ripening (X-axis).

Unique transcripts from each assembled and filtered set were subjected to BLASTx against the non-redundant dataset from NCBI (http://www.ncbi.nlm.nih.gov). From a total of 20,755 unique transcripts from the merged assembly, 92.22% transcripts were annotated while 954 transcripts (4.59%) encoded hypothetical proteins and 661 (3.17%) remained unidentified. BLASTx statistics revealed maximum hits from *Citrus sinensis* and *C. clementina* followed by *Theobroma cacao, Jatropha curcas, Vitis venifera* and *Ricinus communis* (Fig. 1a). Total 74,330 GO terms related to various biological processes (BP), molecular functions (MF) and cellular components (CC) were assigned to these 20,755 unique transcripts. Metabolic, cellular and single organism processes followed by biological regulation and localization were the most abundant terms under the BP category; binding and catalytic activity related terms under MF category; while, organelle, membrane and macromolecular complex terms under the CC category, respectively (Fig. 1b, Supplementary Figure SF1).

Unique transcripts were assigned enzyme commission (EC) number to determine the involvement of these transcripts in various BPs. Total 4,611 ECs were assigned from oxidoreductase, transferase, hydrolase, ligase, lyase and isomerase classes; wherein, transferases were the most abundant followed by hydrolases in all the eight tissues (Fig. 1c). These assigned ECs represented 142 known pathways from the KEGG database (http://www.genome.jp/kegg/pathway.html)^31–33^, which are potentially functional in Alphonso mango fruit development and ripening. Most of these pathways were saturated with higher number of annotations from transcriptome data e.g. metabolism of starch, sucrose and various amino acids including methionine and biosynthesis pathway of ethylene, phenylpropanoids and flavonoids (Supplementary Figure SF2).

### Transcriptome changes through flower to fruit and fruit development to ripening

Variations in the transcriptome were studied using several parameters, such as differentially expressed transcripts, transcripts distinctive to a stage and gene ontology enrichment during flower to fruit transition and through process of fruit development and ripening.

#### Differentially expressed transcripts

Comparison between adjacent tissue stages was carried out to identify differentially expressed transcripts at each stage of fruit development and ripening (Table 3, Supplementary Table ST1). Flower to fruit of 30DAP, 524 transcripts were down regulated while 181 were up-regulated. Among the down-regulated transcripts alpha-amylase and subtilisin inhibitor-like (contig_6593), carbonic anhydrase (contig_5377), chitinase (contig_4907) and maternal effect embryo arrest 59 (contig_4949) exhibited the highest fold change (>2-fold); while homeodomain-like protein (contig_12912 and 12913), cytochrome p450 - cyp72a219-like (contig_4083), heat shock cognate 70 kDa protein (contig_5948) and inositol-3-phosphate synthase (contig_9620) were highly up-regulated (>2-fold).

**Table 3:** Number of differentially expressed transcripts.

Comparison	Up regulated	Down regulated
30DAP vs Flower	181	524
60DAP vs 30DAP	73	7
90DAP pulp vs 60DAP	31	158
90DAP skin vs 60DAP	4	93
90DAP skin vs 90DAP pulp	54	4
5DAH vs 90DAP pulp	42	52
10DAH vs 5DAH	191	418
15DAH vs 10DAH	30	12

Transition from 30 DAP to 60 DAP resulted in up-regulation of 73 and down-regulation of seven transcripts. Important down-regulated transcripts were, n-acetyltransferase (contig_1261), 9-*cis*-epoxycarotenoid dioxygenase (contig_1680), protein reversion-to-ethylene sensitivity (contig_9164) and ethylene receptor 2 (contig_6147). While important up-regulated transcripts were nucleotide sugar transporter family protein (contig_6337), beta-xylosyltransferase (contig_4150), various cellulose synthase catalytic subunits and laccases. Comparison of 60 DAP fruit tissue with 90 DAP pulp and skin tissue, respectively revealed down-regulation of beta-xylosyltransferase, beta-d-xylosidase, cellulose synthase and galacturonosyl transferase; whereas spx and exs domain-containing protein was up-regulated in both 90 DAP pulp and skin. Up-regulation of homeobox protein sbh1, gdsl esterase lipase, caffeoyl shikimate esterase, hydroperoxide lyase, udp-rhamnose:rhamnosyl transferase and pectinesterase inhibitor was evinced from 90 DAP pulp tissue compared to that in 60 DAP fruit tissue. Evaluation of differentially expressed transcripts between pulp and skin of 90 DAP revealed very few transcripts down-regulated in skin with none of them showing change more than 2-fold, while 54 transcripts were up-regulated in 90 DAP skin tissue including the important ones as DNA mismatch repair protein msh5, acyl carrier protein, amino-acid permease, calcium-transporting ATPase, isoflavone reductase and various disease resistance proteins.

During ripening of Alphonso pulp from 90 DAP to 5 DAH, 42 transcripts were up-regulated (>2-fold) including methyltransferase, amino-acid permease, chloroplastic 9-*cis*-epoxycarotenoid dioxygenase, beta-galactosidase and protein phosphatase mainly, whereas 52 transcripts were down-regulated, important ones being few disease resistance proteins, peroxidase, sucrose synthase and glycerol-3-phosphate dehydrogenase. The highest level of differential expression was evinced through transition from 5 DAH to 10 DAH among all the ripening tissues wherein 418 transcripts were down and 191 were up-regulated. These stages are known for non-climacteric to climacteric transition during Alphonso fruit ripening based on metabolite analysis^17, 34^. Prominently down regulated transcripts were phospholipase-A LCAT3, sugar phosphate exchanger, auxin-responsive protein IAA9, abscisate β-glucosyltransferase and membrane-associated kinase regulator with more than 3-fold change, while up-regulated transcripts were aspartic proteinase nepenthesin, UDP-glucose 6-dehydrogenase, 1-aminocyclopropane-1-carboxylate oxidase, peroxidase, bidirectional sugar transporter sweet1-like, omega-6 fatty acid desaturase and squalene monooxygenase. During the transition from 10 DAH to 15 DAH stage, increased phosphate metabolism was evident. Total 30 transcripts were up-regulated mainly including phospholipase-D, inorganic pyrophosphatase, phosphate transporter, transcription factor glk2 and DNA translocase, whereas 12 transcripts were down-regulated and important ones were aspartic protease, plastocyanin-like domain protein, annexin d2-like and glucuronosyltransferase (>2-fold).

#### Idiosyncratic transcripts for the stage

**Figure 2:**
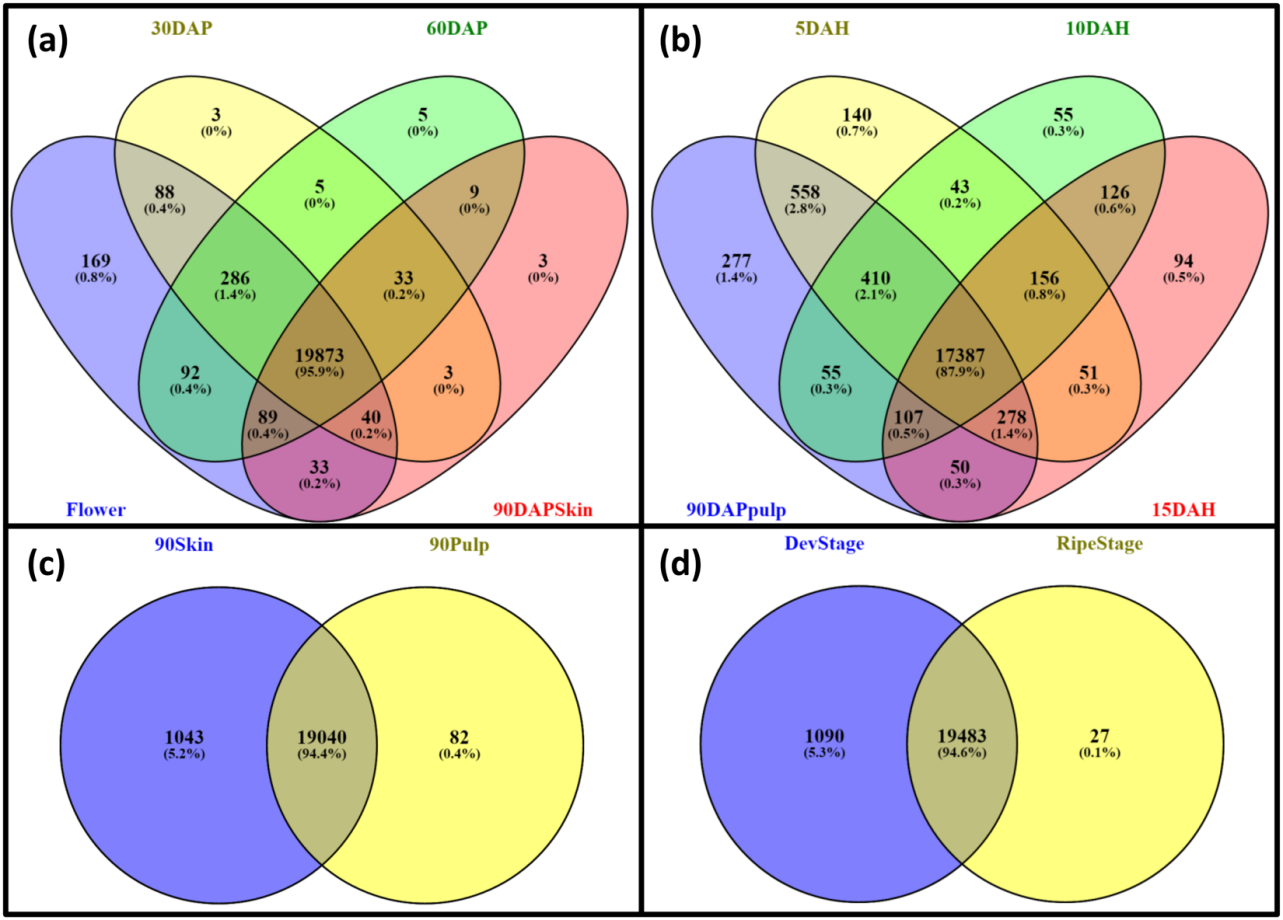
Venn diagrams representing common and distinct transcripts through various comparisons (**a**) Flower, 30 DAP, 60 DAP and 90 DAP skin; (**b**) 90 DAP pulp, 5 DAH, 10 DAH and 15 DAH; (**c**) 90 DAP pulp and 90 DAP skin and (**d**) fruit developing and ripening stages.

Various transcripts unique to each of these stages (Fig. 2, Table 4, Supplementary Table ST2) representing multiple stage specific processes were identified during this comparative analysis. A total of 388 transcripts were identified as unique to flower tissue, which mostly included various transcription and translation factors, late embryogenesis abundant proteins, stress-sensitive and dehydration-responsive element-binding protein-1b and various ribosomal proteins.

**Table 4:** Distinct transcripts.

Comparison	Stage/s	No. of unique transcripts
30DAP vs Flower	Flower	383
	30DAP	44
60DAP vs 30DAP	30DAP	134
	60DAP	195
90DAP pulp vs 60DAP	60DAP	1306
	90DAP pulp	36
90DAPskin vs 60DAP	60DAP	388
	90DAP skin	79
90DAP skin vs 90DAP pulp	90DAP pulp	82
	90DAP skin	1043
5DAH vs 90DAP pulp	90DAP pulp	489
	5DAH	390
10DAH vs 5DAH	5DAH	1027
	10DAH	343
15DAH vs 10DAH	10DAH	563
	15DAH	473
Devlopment vs Ripening	Development	1090
	Ripening	27

Similarly, distinct transcripts specific to fruit developing and ripening stages were identified, wherein 1,090 and 27 transcripts were idiosyncratic to the fruit developing and ripening stages, respectively (Supplementary Table ST2). Various auxin and gibberellin induced and regulated proteins as well as several proteins responsible for various vacuole activities, multiple disease resistance proteins and various terpene synthases were distinct to the developing stages. Transcripts encoding for multiple transcription and translation factors during Alphonso fruit development were also detected. Interestingly multiple ethylene responsive transcription factors along with the protein reversion-to-ethylene sensitivity were uniquely revealed in the developing stages. Whereas, NIN-like protein, respiratory burst oxidase homolog protein d-like, rhamnogalacturonate lyase b-like, lectin receptor kinase, gag protein and methionine Υ-lyase were exclusive to the Alphonso ripening stages. Similarly, WRKY transcription factor 43, b3 domain-containing val3 and ap2 ERF domain-containing transcription factors were uniquely identified from the ripening stages. Several hypothetical and uncharacterized proteins were also found to be Alphonso ripening specific and their characterization might help to reveal the ripening process in Alphonso.

#### Gene ontology (GO) enrichment

**Figure 3:**
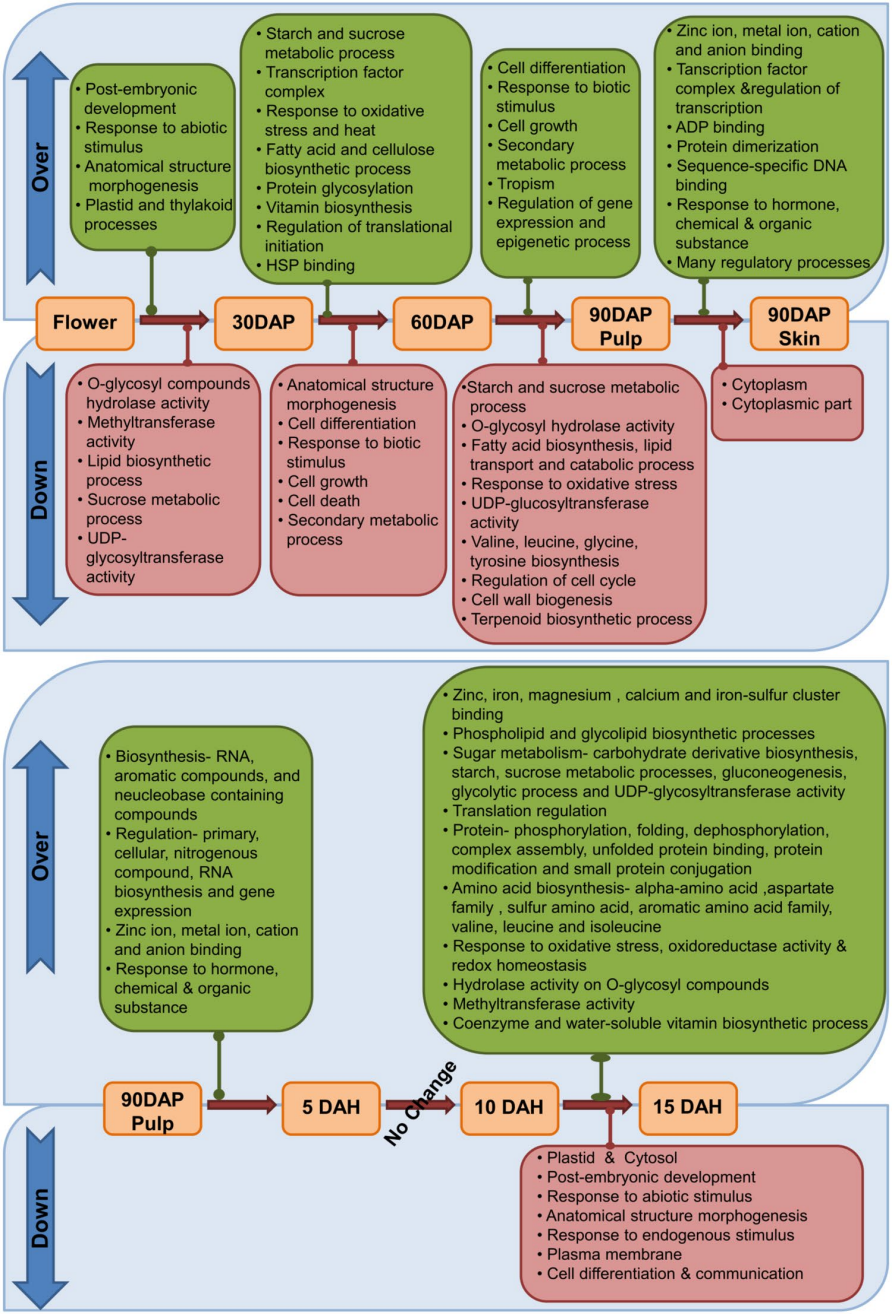
Over and down expressed gene ontologies (GO) between stages of development and ripening.

Fisher’s exact test was performed to understand over- and down-represented GOs (p-value ≤ 0.001) during the transition, which gave an overall picture of the Alphonso mango development and ripening (Fig. 3). During the flower to 30 DAP fruit transition certain GOs were over-expressed such as post-embryonic developmental and anatomical structure morphogenesis process, response to abiotic stimulus and various plastid and thylakoid processes. At the same time, hydrolysis of *O-*glycosyl compounds and UDP-glycosyltransferase activity along with the methyltransferase, sucrose metabolic and lipid biosynthetic activities coding GOs were down-represented. 30 DAP to 60 DAP transition described enriched GOs for starch and sucrose metabolic process, fatty acid, cellulose and vitamin biosynthetic processes, protein glycosylation, response to oxidative stress and heat along with HSP binding. During the same event cell differentiation, growth and cell death in addition to the anatomical structure morphogenesis, secondary metabolic process and response to biotic stimulus coding GOs were observed to be decreased. Comparison between 60 DAP fruit and 90 DAP pulp revealed over-represented GOs for cell differentiation and cell growth, response to biotic stimulus, secondary metabolic process, tropism and regulation of gene expression. While, starch and sucrose metabolic processes, *O-*glycosyl hydrolase UDP-glucosyltransferase activities, fatty acid biosynthesis and catabolic processes along with the lipid transport, various amino acid and terpenoid biosynthesis and cell wall biogenesis were down-represented. 90 DAP pulp and skin tissues showed over-expression of various ion, ADP and sequence-specific DNA binding activities along with the response to hormone, chemical and organic substances in the skin tissue; whereas only cytoplasm and cytoplasmic part related GOs were found to be down-expressed in the skin compared to that in the pulp at 90 DAP.

Transition of 90 DAP pulp to 5 DAH pulp revealed only over-representation of GOs such as biosynthesis of RNA, aromatic compounds, and nucleobase containing compounds; binding of various ions; regulation of primary, cellular, nitrogenous compounds, RNA biosynthesis processes and gene expression and response to hormone, chemical and organic substance. Surprisingly, none of the GOs showed significant (P-value ≤ 0.001) enrichment during the transition from 5 DAH to 10 DAH and reflected as the stationary phase of Alphonso mango ripening. The 10 DAH (mid ripe stage) to 15 DAH (ripe stage) transition showed over-expression of many GOs such as binding of various ions; phospholipid, glycolipid, amino acid, co-enzyme and water soluble vitamin biosynthetic processes; methyltransferase, UDP-glycosyltransferase and *O-*glycosyl hydrolase activities; starch, sucrose metabolic processes, gluconeogenesis and glycolytic process; protein phosphorylation, dephosphorylation, folding, protein modification and small protein conjugation along with the regulation and response to oxidative stress, oxidoreductase activity and redox homeostasis. While the GOs related to plastid and cytosol; post embryonic development; response to abiotic and endogenous stimulus; anatomical structure morphogenesis, plasma membrane, cell differentiation and communication were down-represented.

### Spatial changes in transcriptome at 90 DAP

In case of Alphonso mango, 90 DAP stage is a mature raw stage of the fruit and is considered as the right stage of fruit harvest (0 DAH) for further artificial ripening of the fruit^35^. Hence transcriptome analysis of skin and pulp were separately carried out at this stage. Overall 90 DAP skin was found to be metabolically more active compared to the 90 DAP pulp with respect to differentially expressed genes (Supplementary Table ST1), unique genes (Supplementary Table ST2) and enriched GOs (Fig. 3). In the skin, 54 transcripts were up-regulated whereas only four were down-regulated compared to the pulp. Among the up-regulated transcripts (>2-fold), isoflavone reductase, transcription and translation regulatory proteins, hydrolases, methyltransferase etc. were more prominent; whereas four down-regulated transcripts were membrane and cytoplasm related GOs. Unique transcripts upon comparison between 90 DAP pulp and skin showed carotene and xanthophylls biosynthesis related contigs, beta-carotene hydroxylase (contig_5406) and anthocyanidin 3-o-glucosyltransferase, flavor related various terpene synthases and ripening related contigs such as ethylene-responsive transcription factors, pectate lyase, pectin esterase and cellulase as unique to skin compared to the pulp. Interestingly, in comparison of 5DAH stage with 90 DAP pulp few of these transcripts such as isoflavone reductase (contig_5337) and beta-carotene hydroxylase (contig_5406) were observed to be idiosyncratic to 5 DAH stage as seen in 90 DAP skin stage. Similarly, transcripts encoding enzymes involved in pectin degradation (contigs_9578, 9579, 18096 and 3603) were found to be upregulated (>2-fold) in the comparison between 10DAH vs 90 DAP pulp and 15 DAH vs 90 DAP pulp. These finding suggest progression of ripening related molecular processes in the pulp of 5 DAH and onward stages which were initiated at 90 DAP skin and highlight initiation of Alphonso ripening process from skin and its probable progress towards fruit stone.

### Genes involved in the flavor biogenesis in Alphonso mango

**Figure 4:**
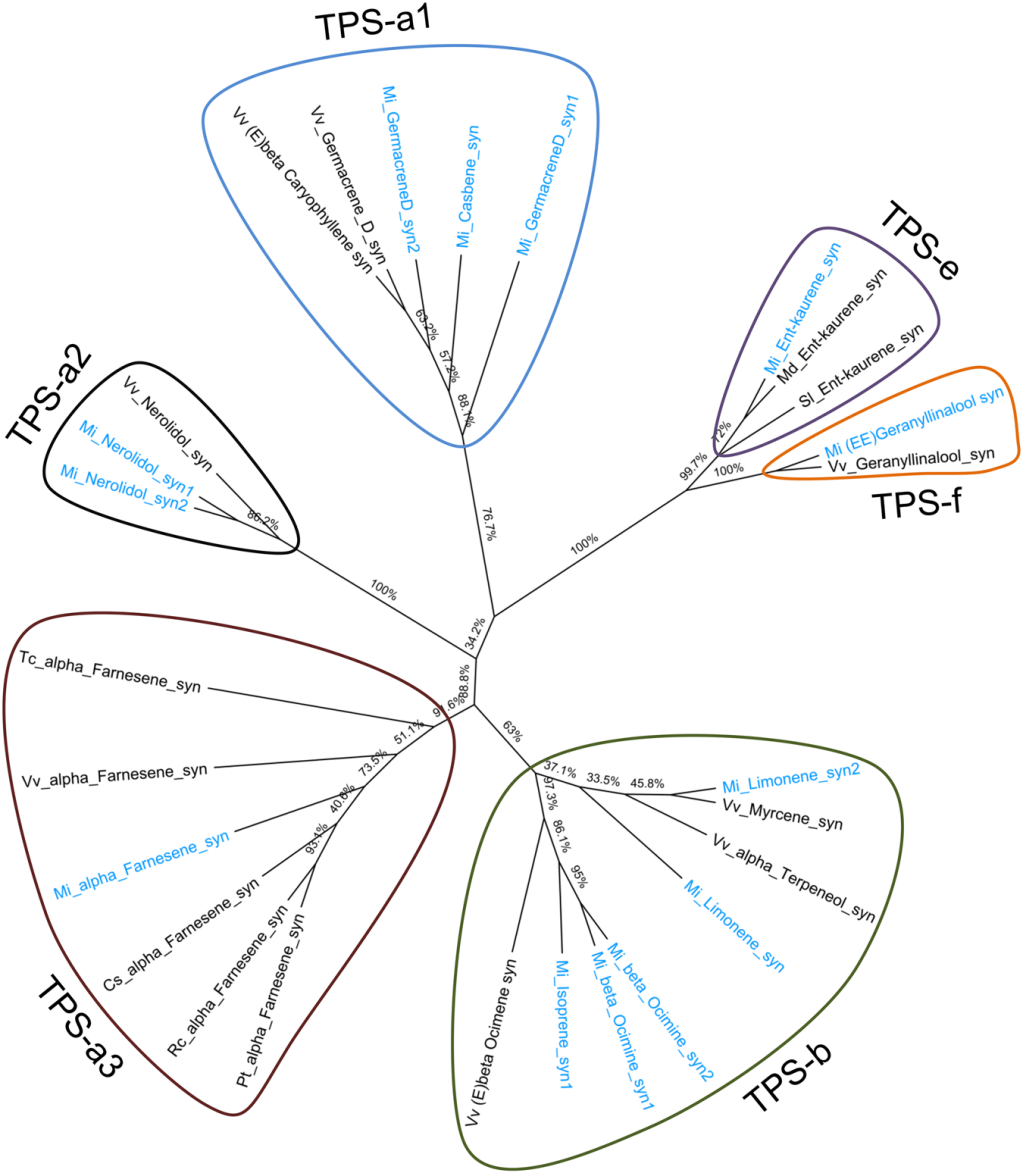
Cladogram representing phylogenetic analysis of proteins encoded by various terpene synthases identified in the present study (blue color) along with the terpene synthases from other Angiosperm plants (black color) using the neighbor-joining method. The node label is composed of two letters representing botanical name of the plant followed by name of the enzyme. Details of the plant species, enzyme and NCBI accession numbers in parenthesis are as follows *Vitis vinifera*_(E)-beta-caryophyllene synthase (ADR74192.1), *Vitis vinifera*_germacrene D synthase (ADR74198.1), *Vitis vinifera*_nerolidol synthase (ADR74211.1), *Vitis vinifera*_(E,E)-geranyl linalool synthase (ADR74219.1), *Malus domestica*_ent-kaurene synthase (AFG18184.1), *Solanum lycopersicum*_ent-kaurene synthase (AEP82778.1), *Vitis vinifera*_(E)-beta-ocimene synthase (ADR74204.1), *Vitis vinifera*_Alphaterpeneol synthase (ADR74202.1), *Vitis vinifera*_myrcene synthase (NP_001268009), *Citrus sinensis*_alpha-farnesene synthase (XP_006467948.1) *Populus trichocarpa*_alpha-farnesene synthase (XP_002317269.2), *Ricinus communis*_alpha-farnesene synthase (XP_015574261.1), *Theobroma cacao*_alpha-farnesene synthase (EOY28527.1) and *Vitis vinifera alpha*_farnesene synthase (NP_001268183.1).

Quantitatively mono-terpenes are abundant in Alphonso followed by sesqui-terpenes^2, 35^. The present data revealed six contigs encoding mono-terpene synthases (*limonene synthase1, limonene synthase2, beta ocimine synthase1, beta ocimine synthase2*, *isoprene* synthase1 and *isoprene synthase2*), five contigs encoding sesquiterpene synthases (*germacreneD synthase1, germacreneD synthase2, nerolidol synthase1, nerolidol synthase2* and *alpha farnesene synthase*) and three contigs coding for di-terpene synthases (*ent-kaurene synthase* and *casbene synthase (E,E)-geranillinalool synthase*). Phylogenetic analysis (Fig. 4) of these genes along with other plant terpene synthases (TPS) showed distribution of these genes in to the TPS-a, TPS-b, TPS-e and TPS-f clades, respectively^36^.

Furaneol and mesifuran are the two furanones from Alphonso mango and their synthesis by *enone oxidoreductase* (*EO*) and *O-methyltransferase* (*OMTS*), respectively have been described earlier^10, 11^. Multiple contigs coding for *quinone oxidoreductase* and *O-methyltransferases* were detected in the present analysis. Phylogenetic analysis of these contigs with the characterized genes revealed another similar transcript variant for the *MiEO* (Supplementary Figure SF3), whereas none of the contigs showed similarity to the *MiOMTS* (Supplementary Figure SF4). Green grassy aroma of unripe fruits is due to the C6 volatiles formed during the lipoxygenase and hydroperoxide lyase (HPL) pathways. In the present study a single contig encoding the *hydroperoxide lyase* and six contigs encoding *13-lipoxygenase* were detected. Involvement of *9-lipoxygenase* (*Mi9LOX*) and epoxide *hydrolase 2* (*MiEH2*) in the biogenesis of lactones from Alphonso mango was confirmed in our recent study^15^. One more transcript encoding *9-lipoxygenase* similar to that of the characterized *Mi9LOX* (Supplementary Figure SF5) and three contigs coding for *epoxide hydrolase 2* grouping with the *MiEH2* (Supplementary Figure SF6) were additionally detected in the present study. Three more novel contigs coding for *epoxide hydrolase* were also detected but neither grouped with the other *EH1*or *EH2* from different plant species (Supplementary Figure SF6).

We further analyzed the differential expression of all these flavor related genes in terms of transcript abundance in flower and fruit developing and ripening stages (Supplementary Figure SF7). Among these the contigs encoding various TPS were abundant in flower and during early developing stages. Interestingly, many contigs encoding EH (contigs 8280, 3904, 3901, 14123 and 8281), LOX (contigs 18105, 12748, 12747 and 12385) and EO (contigs 8026, 5618, 15594, 14137 and 13600) were found to be ripening specific and might be playing crucial role in generating unique aroma volatiles during Alphonso fruit ripening.

### Glycosidases and cell wall degrading enzymes from Alphonso mango

Glycosidases are involved in a variety of functions such as hydrolysis of complex carbohydrates (storage and structural) to mono-saccharides, removal of sugars from various glycans including glycosidically bound aroma volatiles, which serve as storage pool for the aroma compounds. In the present study, many glycosidases acting on various sugars i.e. glucose, galactose, mannose, fructose, xylose, fucose and rhamnose, were detected. Among these, class glucosidase with the highest number of contigs (51 contigs) was observed to contain 28 and 21contigs coding for glucan β-glucosidase and general β-glucosidase, respectively. Two contigs coding for α-glucosidases were identified of which one coded for glucan α-glucosidase and the other for general α-glucosidase. Among these, at 30 DAP stage contig_7442 and contig_7857 were found to be down-regulated (>3-fold) compared to those in flower stage. Contig_16888 was down-regulated whereas contig_17138 was up-regulated at 10 DAH than those at 5 DAH. At 15 DAH contig_9072 was down-regulated (1.5-fold) compared to that at 10 DAH. Among the galactosidase class, nine contigs encoding α-galactosidase and 21 contigs encoding β-galactosidase were detected. Two of these were down-regulated (contig_1095 and contig_1096) at 30 DAP compared to those in flower. Contig_3844 was down regulated in 90 DAP pulp compared to 60 DAP.

Transition from 90 DAP to 5 DAH reflected in to up-regulation of contig_3844 (2.72-fold) whereas, contig_1525 coding for α-galactosidase was down-regulated in 10 DAH compared to that at 5 DAH. In the mannosidase class, five α-mannosidase and eight endo-β-mannosidase coding contigs were identified out of which two encoding for endo-beta-mannosidase (contig_15554 and contig_15558) were down-regulated at 10 DAH compared to 5 DAH, rest didn’t show differential regulation. Among two α-xylosidase and five β-xylosidase from Alphonso mango only contig_1633 showed differential regulation which was up-regulated at 60 DAP compared to 30 DAP and was further down-regulated in both the 90 DAP tissues. Two transcripts coding for the acid beta-fructofuranosidase (contig_12501 and contig_12502) were detected but did not show any differential regulation in various tissues analyzed. Also, seven contigs coding for the alpha-l-fucosidase were identified from Alphonso mango but did not show differential regulation.

Degradation of plant cell wall components, namely cellulose, hemi-cellulose and pectin by cellulases and glucanases in ripening fruits is responsible for the fruit softness. Cellulases encoding five contigs were identified among which one coded for acidic cellulase and found to be abundant during flower and early fruit developing stages. Total 18 contigs encoding glucanase were detected of which contig_4148 was up-regulated in flower compared to 30 DAP, whereas, four transcripts (contig_9268, contig_19283, contig_9267 and contig_17145) were down-regulated (>2-fold) at 10 DAH compared to 5 DAH stage. Pectin is another component of fruit cell wall and is degraded by a set of enzymes *viz*. pectate lyase (PL), pectin esterase (PE), polygalacturonase (PG) and rhamnogalacturonate lyase. In the present study 18 PL, 24 PG and 10 PE coding transcripts were detected. However, only few were differentially expressed, for example, only PL contig_7696 was down-regulated in 30 DAP fruit compared to flower and PL contig_9578 was up-regulated in 10 DAH fruit than 5 DAH fruit. PG non-catalytic subunit jp650 coding contig_9895 was down-regulated in 5 DAH pulp than in 90 DAP pulp, whereas PG contig_3471 was down regulated and PG contig_1614 was up-regulated in 10 DAH compared to 5 DAH stage. Likewise, PE contig_7997 was down-regulated and contig_9162 was up-regulated at 30 DAP stage compared to flower. Similarly, eight transcripts encoding rhamnogalacturonate lyase were detected of which two (contig_7745 and contig_7746) were distinct to the ripening stages (Supplementary Table ST2).

### Transcriptome analysis identified novel enzyme inhibitors from Alphonso mango

We identified various classes of enzyme inhibitors (Supplementary Table ST3) such as α- amylase inhibitor (2 contigs), inhibitor of proliferation pds5, apoptosis inhibitor (2 contigs), bax inhibitor (4 contigs), lipid transfer protein inhibitor (6 contigs), various kinase inhibitors (16 contigs), cysteine proteinases inhibitor (4 contigs), inter-alpha-trypsin inhibitor (3 contigs), serine protease inhibitor, kunitz family trypsin and protease inhibitor, guanosine nucleotide diphosphate dissociation inhibitor, nf-kappa-b inhibitor (2 contigs), pectinesterase inhibitor (12 contigs), polygalacturonase inhibitor, proteasome inhibitor, protein transport inhibitor (4 contigs), protein phosphatase inhibitor and rho gdp-dissociation protein inhibitor (4 contigs). Along with these, contigs coding for protein reversion-to-ethylene sensitivity (3 contigs), cell wall and vascular inhibitor of beta-fructosidase (2 contigs) and macrophage migration inhibitory factor were also identified from Alphonso transcriptome. These inhibitors showed differential regulation during fruit development and ripening (Supplementary Figure SF8). Most of the inhibitors were found to be expressing throughout all the stages except for the group 4 (coding for bax inhibitor), which were abundant only during fruit ripening stages of Alphonso mango and probably played important role in ripening physiology of Alphonso mango.

### Transcriptome validation through qRT PCR

**Figure 5:**
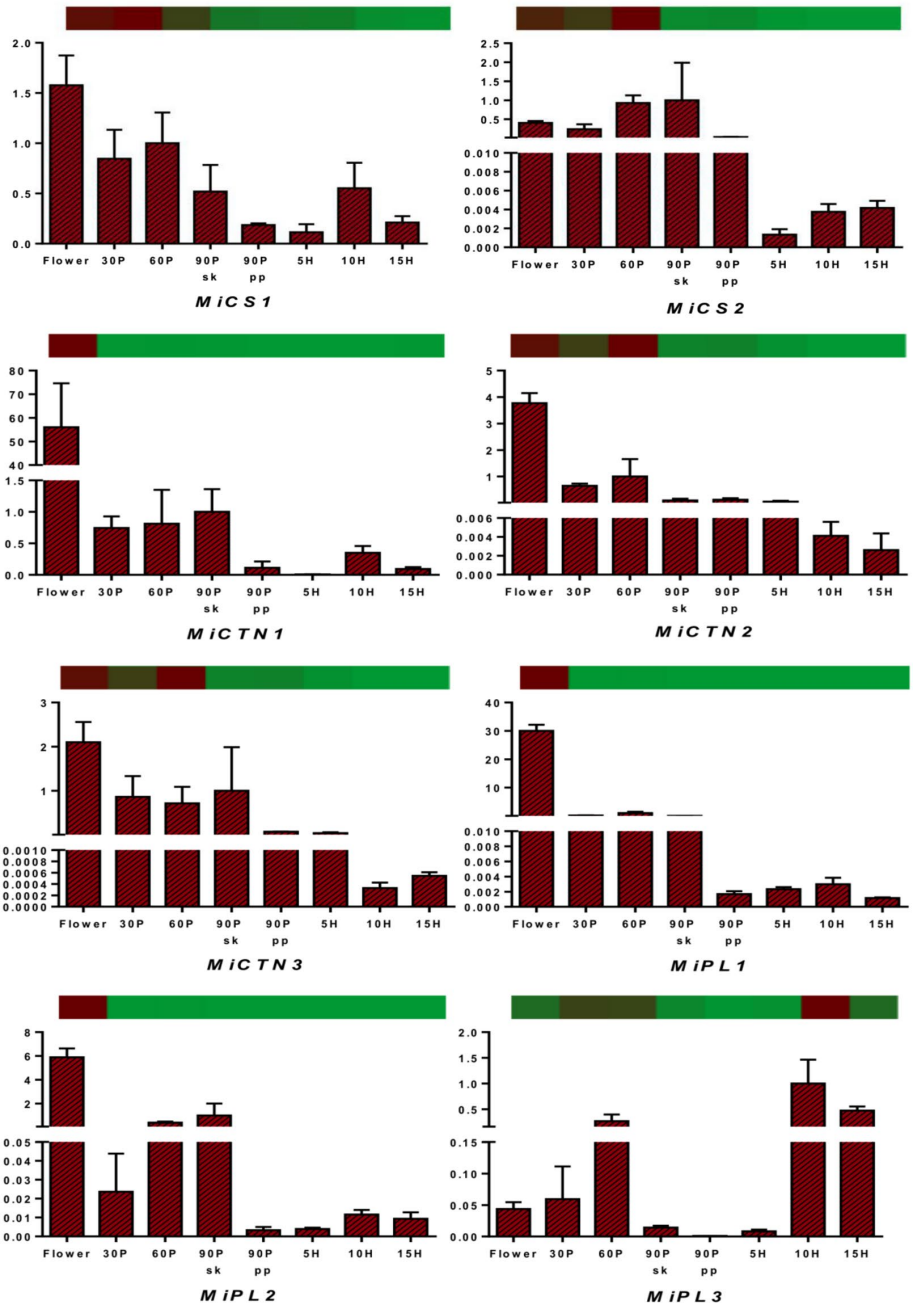
Quantitative reverse-transcriptase PCR validation of various transcripts obtained through RNAseq from carbohydrate metabolism through various tissues (flower and fruit developing and ripening stages). Vertical bars at each data point represent standard error in the relative quantification among the three biological replicates. X-axis represents fruit development and ripening stages and Y-axis represents relative transcript abundance. The horizontal bar above each histogram represents the expression level of the same transcript as obtained through RNAseq analysis, wherein dark-red color indicates higher expression and light-green color indicates lower expression. Gene names are as referred in result section “Transcriptome validation through qRT PCR”.

**Figure 6:**
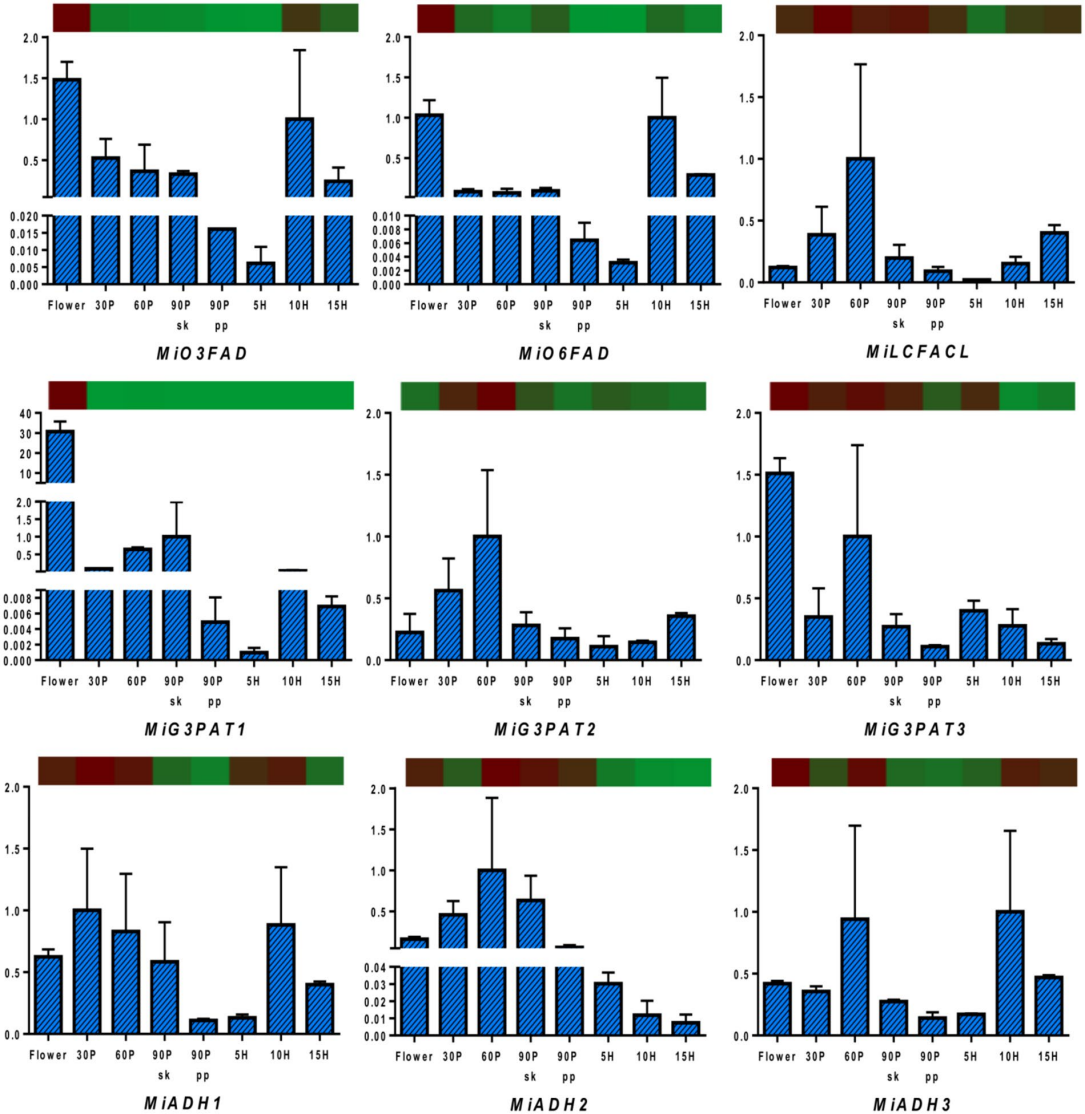
Quantitative reverse-transcriptase PCR validation of various transcripts obtained through RNAseq from lipid metabolism through various tissues (flower and fruit developing and ripening stages). Vertical bars at each data point represent standard error in the relative quantification among the three biological replicates. X-axis represents fruit development and ripening stages and Y-axis represents relative transcript abundance. The horizontal bar above each histogram represents the expression level of the same transcript as obtained through RNAseq analysis, wherein dark-red color indicates higher expression and light-green color indicates lower expression. ﻿Gene names are as referred in result section “Transcriptome validation through qRT PCR”.

**Figure 7:**
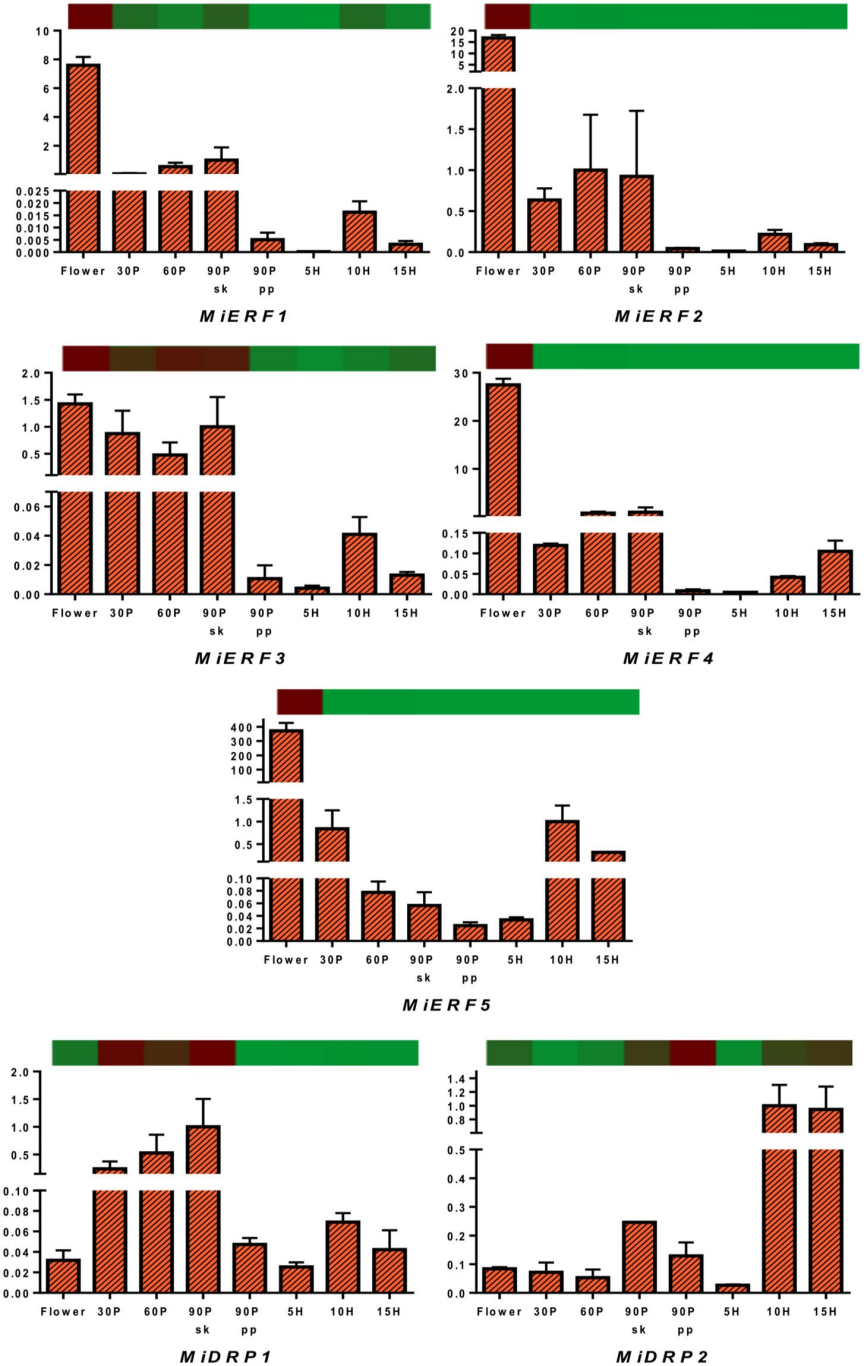
Quantitative reverse-transcriptase PCR validation of various transcripts obtained through RNAseq from ethylene responsive factors (ERF) and disease resistance proteins (DRP) through various tissues (flower and fruit developing and ripening stages). Vertical bars at each data point represent standard error in the relative quantification among the three biological replicates. X-axis represents fruit development and ripening stages and Y-axis represents relative transcript abundance. The horizontal bar above each histogram represents the expression level of the same transcript as obtained through RNAseq analysis, wherein dark-red color indicates higher expression and light-green color indicates lower expression.

**Figure 8:**
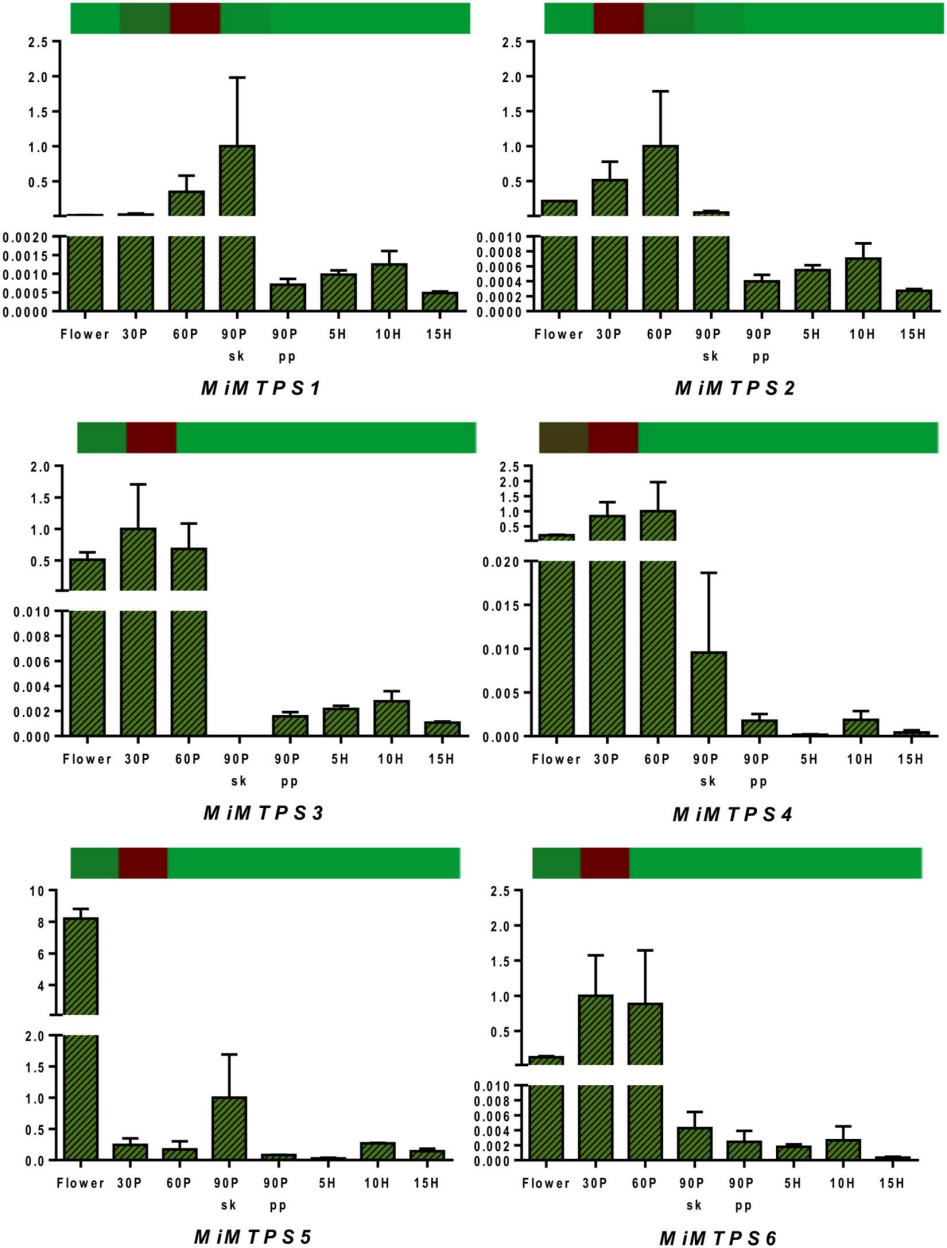
Quantitative reverse-transcriptase PCR validation of various transcripts obtained through RNAseq from mono-terpene metabolism  through various tissues (flower and fruit developing and ripening stages). Vertical bars at each data point represent standard error in the relative quantification among the three biological replicates. X-axis represents fruit development and ripening stages and Y-axis represents relative transcript abundance. The horizontal bar above each histogram represents the expression level of the same transcript as obtained through RNAseq analysis, wherein dark-red color indicates higher expression and light-green color indicates lower expression. ﻿Gene names are as referred in result section “Transcriptome validation through qRT PCR”.

**Figure 9:**
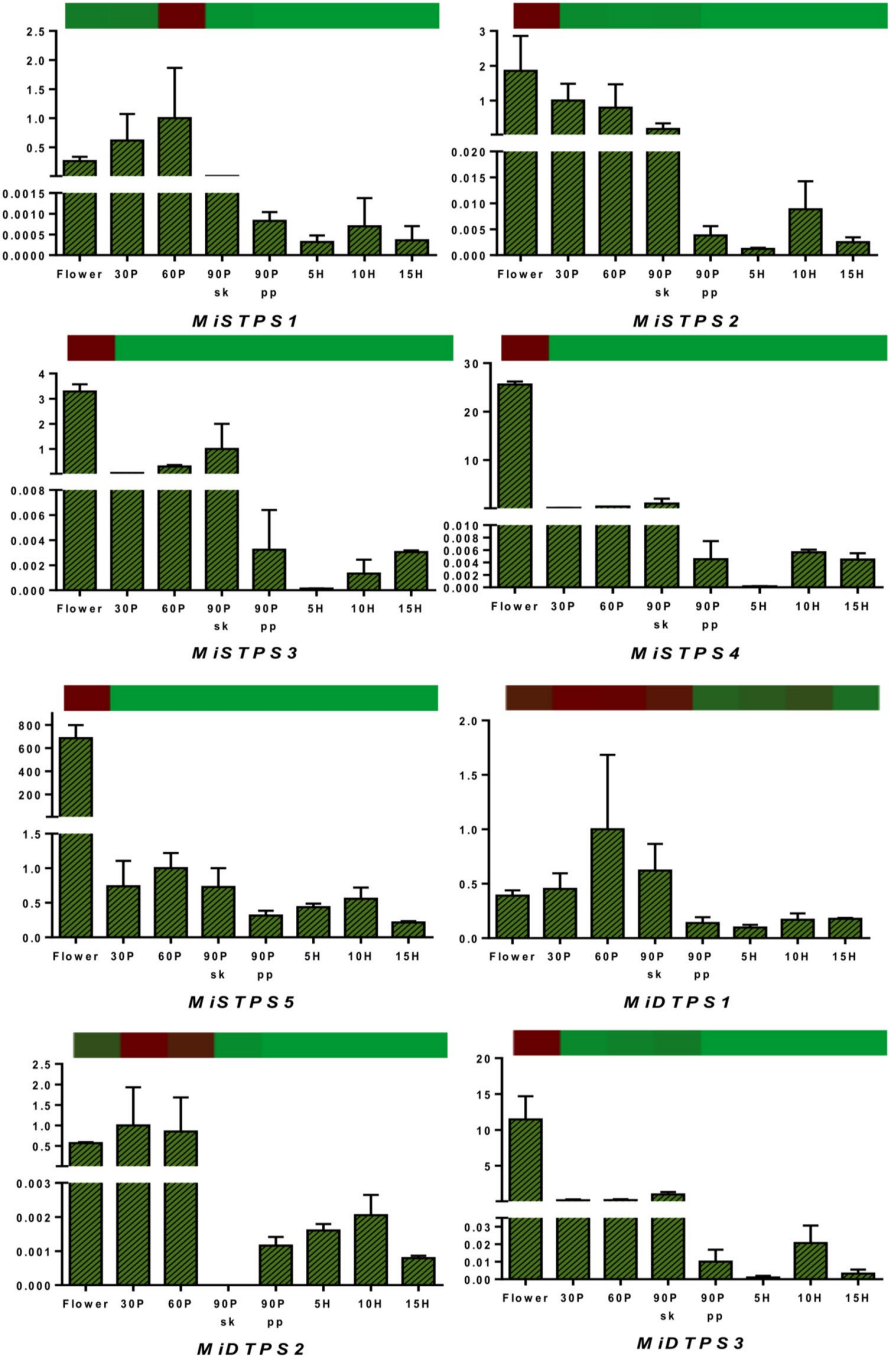
Quantitative reverse-transcriptase PCR validation of various transcripts obtained through RNAseq from sesqui-terpene and di-terpene metabolism   through various tissues (flower and fruit developing and ripening stages). Vertical bars at each data point represent standard error in the relative quantification among the three biological replicates. X-axis represents fruit development and ripening stages and Y-axis represents relative transcript abundance. The horizontal bar above each histogram represents the expression level of the same transcript as obtained through RNAseq analysis, wherein dark-red color indicates higher expression and light-green color indicates lower expression. ﻿Gene names are as referred in result section “Transcriptome validation through qRT PCR”.

Results of the transcriptomic analysis were validated by qRT PCR of 38 representative genes from various metabolic pathways such as carbohydrate metabolism (cellulose synthase: CS, chitinase: CTN, and pectate lyase: PL), fatty acid metabolism (omega 3 fatty acyl desaturase: O3FAD, omega 6 fatty acyl desaturase: O6FAD, glyceraldehyde-3-phosphate acyl transferase: G3PAT, alcohol dehydrogenase: ADH and long chain fatty acyl CoA ligase: LCFACL), terpene metabolism (mono-terpene synthases: MTPS, sesqui-terpene synthases: STPS and di-terpene synthases: DTPS) and proteins such as ethylene responsive factors: ERF and disease resistance proteins: DRP. Various transcript variants of these genes were selected wherever available to confirm the accuracy of assembly. qRT PCR analysis revealed similar differential expression pattern for almost all the transcripts through all the eight stages with correlation coefficient *R* ranging between 0.8 and 0.99. Few transcripts showed variation in the fold change of RNAseq and qRT PCR data at few stages but still had good correlation (*R* ≥ 0.7) (Figs 5–9 and Supplementary Table ST5). Transcript variants confirmed the accurate assembly and showed differential expression of these variants from each other through various tissues analyzed (Figs 5–9).

## Discussion

Recently transcriptome studies on mango cultivars namely, Zill^25^, Langra^26^, Kent^27^ and Dashehari^28^ have put forth important information regarding the fruit and leaf physiology of mango. These studies have identified genes encoding multiple enzymes involved in various pathways of primary and secondary metabolism such as, citrate cycle, glycolysis and gluconeogenesis from carbohydrate metabolism; fatty acid biosynthesis, beta oxidation and salicylic acid biosynthesis from fatty acid metabolism; biosynthesis and degradation of various amino acids as well as ethylene biosynthesis from methionine. Genes involved in the flavonoid biosynthesis, vitamin biosynthesis (β-carotene and α-tocopherols) as well as terpenoid backbone synthesis (mevalonate pathway) have also been well explored. In the present study genes involved in all these pathways were identified and their differential expression was also evident through various stages of the Alphonso mango fruit development and ripening (Supplementary Table ST1). In addition the present study revealed some novel findings highlighting better understanding of various processes involved in mango fruit development and ripening and some were unique to the most favoured Alphonso mango fruit, which are discussed below.

Quantitative abundance of terpenes in mangos is well known^2, 35^, Transcriptome and gene expression studies in mango have explored the terpene biosynthesis pathway till GPP, FPP and GGPP synthesis^9, 25–28^. In this study, we identified six, five and three genes encoding mono-terpene synthases (MTPS), sesqui- terpene synthases (STPS) and di-terpene synthases (DTPS), respectively that are involved in biosynthesis of specific terpene molecules (Supplementary Figure SF9). These genes were abundant in the flower tissue followed by 30 DAP stage. Further, the transcript abundance of many of these terpene synthase genes in the present study has been depicted to be idiosyncratic to the developing stages, leading to their least expression in the ripening stages of Alphonso fruit (Figs 8 and 9, Supplementary Figure SF7 and Supplementary Table ST2). Previous aroma volatile analysis from Alphonso mango supports this observation wherein flower had the highest concentration of mono-terpenes, oxygenated mono-terpenes and sesqui-terpenes which decreased through the fruit development^35^.

Another flavor related pathway is lipoxygenase (LOX) followed by HPL pathway^37–39^, which produces C6 GLVs and lactones through peroxygenase pathway^15^. The transcriptomes analyzed from Kent and Dashehari mangos reported the presence of six and five genes coding for the LOX family, respectively^27, 28^. Here, we report detailed annotation of Alphonso mango *LOX* genes wherein, two genes encode the *9-LOX* and six genes encode the *13-LOX*. Likewise, *Peroxygenase* and *epoxide hydrolase* (*EH*) genes have been well-studied for biosynthesis of cutin biopolymer^40^ and defense related compounds^41, 42^; while our recent study has shown involvement of these genes in the production of lactones^15^. In spite of their biological significance, none of the previous transcriptome studies on mango could determine the presence of peroxygenase and epoxide hydrolase genes. On the other hand in the current study various contigs encoding novel EH (three contigs), EH2 (four contigs) and peroxygenase (three contigs) were detected. Similarly, multiple transcripts encoding *enone oxidoreductase* and *O-methyltransferase* having role in furanone biosynthesis^10, 11^ were also identified and expression profiles of many of them were shown to be ripening specific (Supplementary Figure SF7). Abundance and ripening related expression of large number of these unique flavor related genes in Alphonso mango (Supplementary Figure SF7) signifies their role in synthesis of diverse aroma volatiles and their unique blend giving sweet and fruity flavor in Alphonso as shown by our previous studies^34, 35^.

Fruit ripening is a complex physiological process and can be characterized by means of fruit softening due to changes in the cell wall structure^43–45^, increased sugar content by polysaccharide hydrolysis^16^ and changes in the aroma volatiles^35^. Starch and pectin are the major storage and structural polysaccharides in the mango fruit, respectively. Various hydrolases and lyases are known to carry out polysaccharide and cell wall hydrolysis^43, 46–50^, while amylases degrade the starch in to soluble sugars during ripening. We identified four α-amylase, three isoamylase and 13 β-amylase coding transcripts. Only one transcript coding for α-amylase (contig_1439) was down-regulated through flower to 30 DAP fruit transition while others were expressed throughout the fruit developing and ripening stages. On the other hand, transcriptome analysis of Kent mango revealed identification of four β-amylase and three α-amylase transcripts out of which only two β-amylase coding transcripts were found to be up-regulated in ripe tissue, none of the other showed differential expression^27^.

In addition, we also detected large number of PL, PG and PE encoding transcripts known to be responsible for degradation of complex hetero-polysaccharide, pectin^48^ were detected (Supplementary Table ST4). Most of these had stable expression and only few were differentially expressed in Alphonso mango. These results are in contrast to the observations from Kent^27^ and Dashehari^28^ mango transcriptomic data in terms of the number of unigenes detected and their differential regulation. In case of Dashehari mango four PL and none of the PE or PG encoding transcripts were up-regulated; whereas in Kent, four PL, six PE and six PG encoding unigenes were up-regulated. These results signify controlled and steady activity of pectin degradation leading to slow and balanced transitions during Alphonso fruit ripening and this might be one of the reasons for its longer shelf life.

Another interesting observation in our study was, the identification of 79 contigs encoding 20 different inhibitor classes from Alphonso mango (Supplementary Figure SF8). Previously, only three unigenes coding for the cysteine proteinase inhibitors were reported from Langra leaf transcriptome, while no such inhibitors were reported from Zill^25^, Kent^27^ and Dashehari^28^ mango transcriptomes. The present study suggests Alphonso mango transcriptome to be rich in these inhibitors throughout the fruit development and ripening stages.

**Figure 10:**
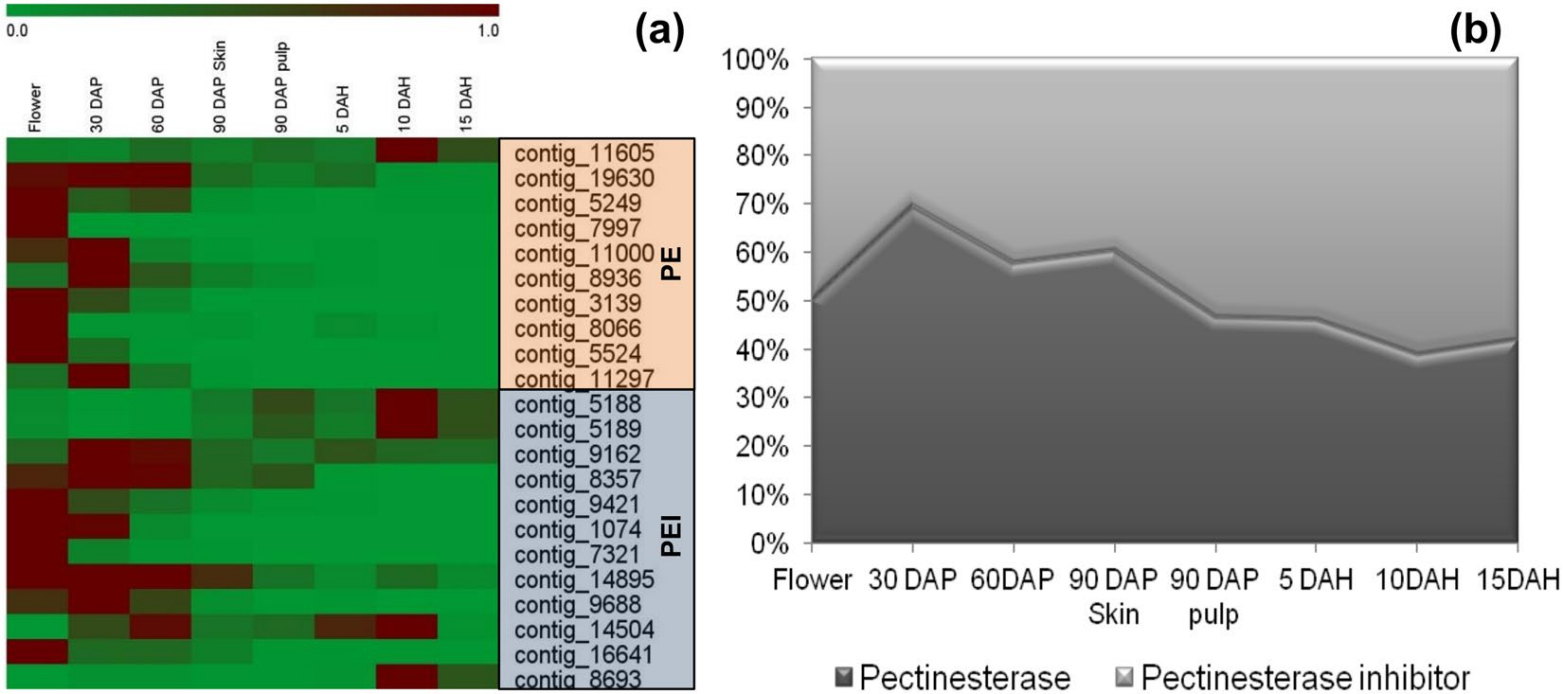
(**a**) Heatmap representing expression profiles of contigs encoding pectinesterase (PE) and pectinesterase inhibitor (PEI) from RNAseq data. (**b**) Representation of percent co-expression (Y-axis) of 10 contigs encoding pectinesterase and 12 contigs encoding pectinesterase inhibitor, respectively by calculating sum of their mapped raw reads in various tissues of Alphonso mango (X-axis).

Thus, the presence of large numbers of amylase, PL, PE and PG transcripts with very few of them differentially regulated, perpetual expression of most of the starch and cell wall hydrolyzing enzymes along with the persistent presence of inhibitors for amylase, pectinesterase, polygalacturonase and ethylene sensitivity can be cumulatively suggested to play a crucial role in controlled and slow ripening and longer fruit shelf life of Alphonso mango. As a representative example the balance between pectinesterases and their inhibitors during Alphonso fruit development and ripening has been demonstrated in Fig. 10 as heatmap and area chart of expression of PE and PEI. We further observed that phosphate metabolism related gene ontologies were enriched at complete ripe stage (15 DAH) of Alphonso mango. Among these some transcripts were uniquely found at 15 DAH stage (Supplementary Table ST2) suggesting accelerated primary metabolism. Moreover, contig 6946 encoding cytokinin riboside 5 -monophosphate phosphoribohydrolase was uniquely observed at 15 DAH stage. This enzyme plays important role in converting endogenous inactive cytokinin nucleotides to the biologically active free cytokinin responsible for delayed ripening^51, 52^. On the other hand contig_6770 encoding phospholipase-D (PLD) involved in degradation of important cell membrane component i.e. phospholipids was observed to be upregulated (3.4fold). A key role of PLD in softening was well explained in tomato plants transformed with antisense PLD cDNA which showed delayed ripening and increased firmness in tomato fruits^53^. This counter play at molecular level might be responsible for fine tuning of ripening process in Alphonso mango providing longer shelf life to the fruits.

Additionally oxidative burst, oxidoreductase activities and oxidative stress related gene ontologies were observed during ripening. These factors can generate reactive oxygen species and lead to cell death and fruit damage. Such reactive oxygen species induced cell death is suppressed by Bax inhibitor, which was well studied in *Arabidopsis thaliana* ^54^. Interestingly, four contigs coding for Bax inhibitor in Alphonso mango showed ripening specific expression probably responsible for preventing cell death in ripening fruits and thus longer shelf life. Further detailed study on these inhibitors might help to understand jelly formation in Dashehari mangos due to excessive ripening and spongy tissue formation in Alphonso mangos due to uneven ripening^55, 56^.

It is known that ripened fruits are prone to attack by various pathogens^57^. A well distinct defense mechanism was observed in Alphonso mango wherein various defense related proteins (227 contigs) and chitinases (19 contigs) acting on fungal cell wall^58^ were differentially regulated (Supplementary Table ST1). Chitinases were found to be accumulated in the flower and in the early fruit developing stages. Insect driven pollination has the risk of fungal infection to flower and further spore accumulation around ovary causing internal infection to the fruit. This might be restricted by the presence of various chitinases in Alphonso mango. Similarly, various disease resistance proteins might play role during fruit development and ripening process to defend infections.

## Conclusions

The transcriptome of Alphonso mango analyzed through eight stages of flower to fruit development and ripening transitions revealed various differentially regulated and stage specific genes. Unique transcript profiles probably responsible for distinct and favorable characteristics of Alphonso mango fruit such as flavor, color, ripening duration, skin to stone ripening pattern and longer shelf life were identified and analyzed. This study provides large data sets for further functional validation of fruit ripening process.

## Methods

### Plant material

Flower and fruits of cv. Alphonso were collected from three independent trees as biological replicates from the Mango Research Sub Centre, Deogad (16.528336 N, 73.344790 E) affiliated to Dr. Balasaheb Sawant Konkan Agricultural University, Dapoli, Maharashtra, India. Flowers from inflorescence were collected and snap frozen. Fruits from developing stages were collected at 30, 60 and 90 days after pollination (DAP). Fruits from 30 and 60 DAP were analyzed as whole fruit in the present study after removing fruit stone; whereas at mature raw stage (90 DAP) pulp (mesocarp) and skin (exocarp) were separated, snap frozen in liquid nitrogen and stored at −80 °C until further analysis. A set of fruits were additionally harvested at their mature raw stage and kept in the hay containing boxes at ambient temperature for ripening and only pulp tissue for ripening stages as table green, mid ripe and ripe were collected at 5, 10 and 15 days after harvest (DAH), respectively. At each ripening stage fruits were removed from the box, pulp and skin were separated and pulp was frozen in liquid nitrogen and stored at −80 °C till further use.

### RNA isolation and cDNA synthesis

Total RNA isolation was carried out for all the tissues sampled for current study using RNeasy Plus mini kit (Quiagen, Hilden, Germany). RNA quality as 260 nm/ 280 nm ratio was evaluated using Nanodrop 1000 (Thermo Fisher Scientific, Waltham, Massachusetts, USA) and RNA integrity was checked using Bioanalyzer 2100 (Agilent Technologies, Santa Clara, USA). Two microgram of total RNA was used to carry out reverse transcription for synthesis of cDNA using High Capacity cDNA reverse transcription kit (Applied Biosystem, Carlsbad, CA, USA).

### Library preparation and sequencing

High quality RNAs from seven progressive stages of fruit development and ripening as well as flower tissue from single representative biological replicate were sent to the Next Generation Genomics Facility (NGGF) at the Centre for Cellular and Molecular Platforms (C-CAMP), Bangalore for performing transcriptome sequencing. Briefly, 1 ug of total RNA from each sample was used to prepare eight individual libraries and mRNA was purified using polyT oligo beads. The purified mRNA was fragmented in the range of 100 to 140 bases with optimum at around 120 bases and the cDNA was synthesized. End repair, A-Tailing, Adapter ligation and the library preparation were performed using Tru Seq RNA sample preparation kit v2 (Illumina, San Diego, USA) as per manufacturer’s instructions. PCR enrichment was performed for 15 cycles and the sample was validated on the Bioanalyzer 2100. Libraries were sequenced in a Paired End 100 base run, using TruSeq SBS Kit v3-HS (Catalog No.: FC-401-3001) for sequencing on the Illumina HiSeq. 1000 platform according to the manufacturer’s recommended protocols. (http://www.illumina.com/systems/hiseq_systems/hiseq_2000_1000/kits.ilmn).

### Bioinformatics data analysis

Paired end RNA sequencing was performed using Illumina Hiseq2000. Quality check on raw data was performed using FastQC (http://www.bioinformatics.babraham.ac.uk/projects/fastqc/). Adapter free, good quality reads (Q >= 30; min read length = 85) were obtained using Cutadapt^59^. Alphonso transcriptome for each stage was assembled using Velvet-Oases^60^ with K-mers 67, 75, 83 and merging them at 27 k-mer. Additionally, a merged transcriptome was also generated using k-mer 55. Further, for all the merged assembled transcripts, we used Transdecoder (https://transdecoder.github.io/) to extract potential candidate coding regions within transcripts. Partial cds were discarded and only those transcripts with start and stop codon were made non redundant based upon sequence identity cut-off of 90% using CD-HIT-est^61^ and used for downstream analyses.

Further, merged full length transcripts (from merged assembly) were used as reference to map back all the raw reads from each stage using default parameters of Bowtie^62^. DESeq2 was used to identify differentially expressed transcripts^63^ and were filtered based upon p-value ≤ 0.05 and expression value >0. All those transcripts having mapping count zero were excluded from further analysis such as in identification of uniquely expressed transcripts in a particular stage or a specific set of stages (e.g. developing and ripening stages). Unique and common list of transcripts were represented using Venny (http://bioinfogp.cnb.csic.es/tools/venny/) and provided in Supplementary Table ST2. Full length transcripts (from merged assembly) were used as reference for differential expression analysis using DESeq2^63^.

Annotation, enzyme code distribution and GO mapping and InterProScan analyses were carried using BLAST2GO 3.1.3 workbench (Biobam Bioinformatics S.L., Valencia, Spain) as described in the user manual^64^. GO enrichment analysis was carried out in given test and reference sets by Fisher’s exact test in BLAST2GO with p-value (0.001) and FDR filters.

### Quantitative reverse-transcriptase PCR

Quantitative reverse-transcriptase PCR was performed using the Fast Start Universal SYBR Green master mix (Roche Inc. Indianapolis, Indiana, USA) and *elongation factor 1α* (*EF1α*) as an endogenous reference gene employing the primers reported earlier^7^. Various transcripts selected from transcriptome data were amplified using gene specific primers (Supplementary Table ST5) and quantification was performed using 7900HT Fast Real-Time PCR System (Applied Biosystems, California, USA) having thermal cycle program of initial denaturation at 95 °C for 10 min with subsequent 40 cycles of 95 °C for 3 sec and 60 °C for 30 sec followed by a dissociation curve analysis of transcripts. Relative quantification (ΔΔCT method) and statistical analysis was carried out using DataAssist™ v3.01 software (Applied Biosystems, California, USA). Eight tissue samples as described above from three independent biological replicates were used for this analysis. For individual transcript the highest expression at a particular fruit development or ripening stage was considered as 1 and expression in other tissues including flower was normalized to that for better graphical presentation.

### Data availability

The raw data files generated during the current study will be available in the NCBI repository under the BioProject - PRJNA391381.

## Electronic supplementary material

Supplementary information

Supplementary Table ST1

Supplementary Table ST2

Supplementary Table ST3

Supplementary Table ST4

Supplementary Table ST5
