# Supplementary material for: Transcriptional transitions in Alphonso mango (Mangifera indica L.) during fruit development and ripening explain its distinct aroma and shelf life characteristics
Source: Sci Rep. 2017 Aug 18;7:8711. doi: 10.1038/s41598-017-08499-5 (PMC5562913; doi:10.1038/s41598-017-08499-5)

Transcriptional transitions in Alphonso mango (*Mangifera indica* L.) during fruit development and ripening explain distinct aroma and shelf life characteristics

Ashish B. Deshpande[a], Krishanpal Anamika [b], Vineet Jha[b], Hemangi G. Chidley[a], Pranjali S. Oak[a], Narendra Y. Kadoo[a], Keshav H. Pujari[c], Ashok P. Giri[a] and Vidya S. Gupta[a,*]

[a] Plant Molecular Biology Group, Biochemical Sciences Division, CSIR-National Chemical Laboratory, Pune-411008, Maharashtra, India

[b] Labs, Persistent Systems Limited, Pingala-Aryabhata, Erandwane, Pune 411004,

[c] Dr. Balasaheb Sawant Konkan Agriculture University, Dapoli-415712, Maharashtra, India

*Corresponding Author

Dr. (Mrs.) Vidya S. Gupta
Plant Molecular Biology Group
Biochemical Sciences Division
CSIR-National Chemical Laboratory
Pune-411008, India
Phone: +91-20-25902247
Fax: +91-20-25902648
E mail: vs.gupta@ncl.res.in

Supplementary Figure SF1: Heatmap representing expression profiles of various gene ontologies from biological process (BP), molecular function (MF) and cellular components (CC) in different tissues, (a) Stage wise normalization and (b) global normalization for each GO category presented.

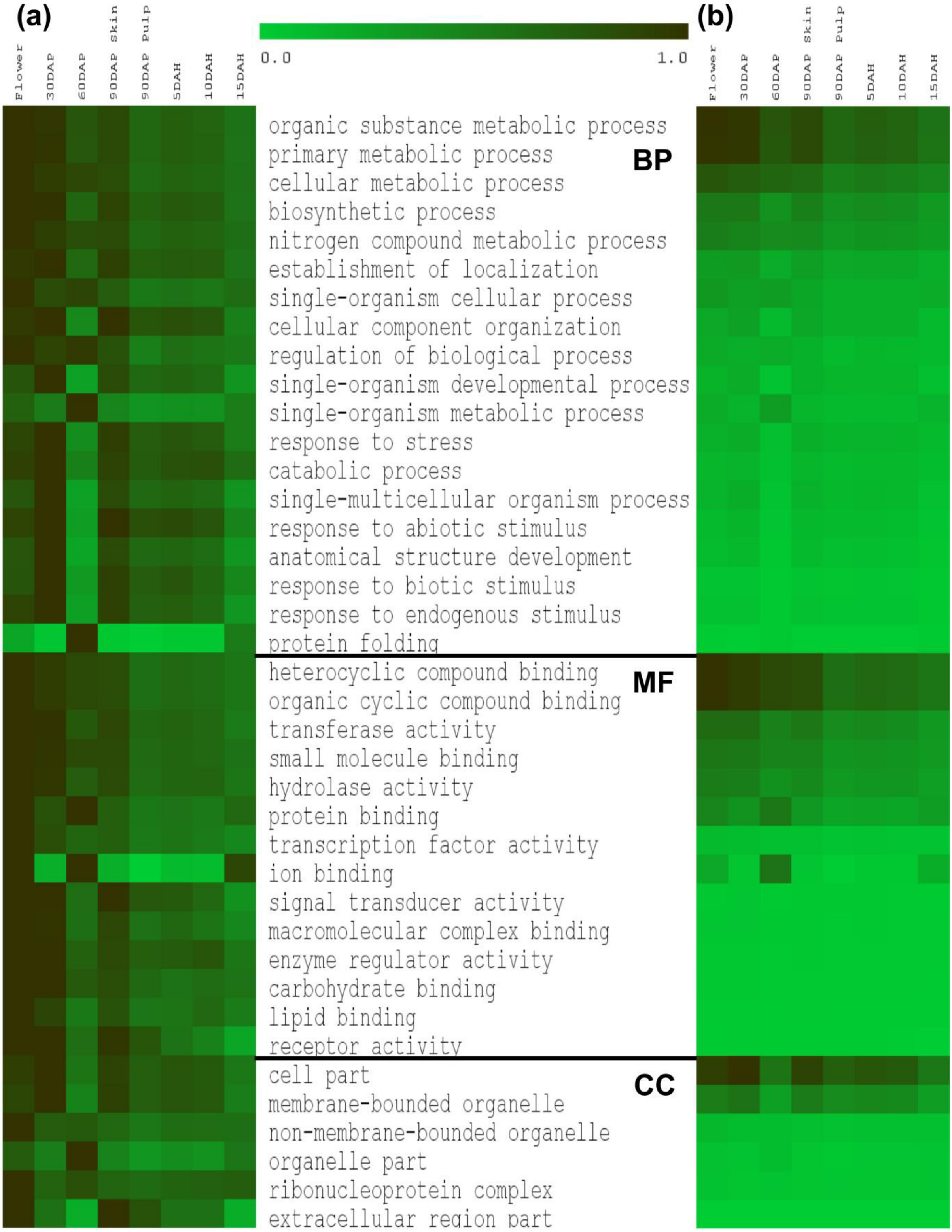

Supplementary Figure SF2: Important pathways, such as (a) starch and sucrose metabolism, (b) cysteine and methionine metabolism and ethylene biosynthesis, (c) phenylpropanoid biosynthesis and(d) flavonoid biosynthesis, which are functional in Alphonso mango. Data sourced from the KEGG pathway database (Kanehisa laboratories).

(a)

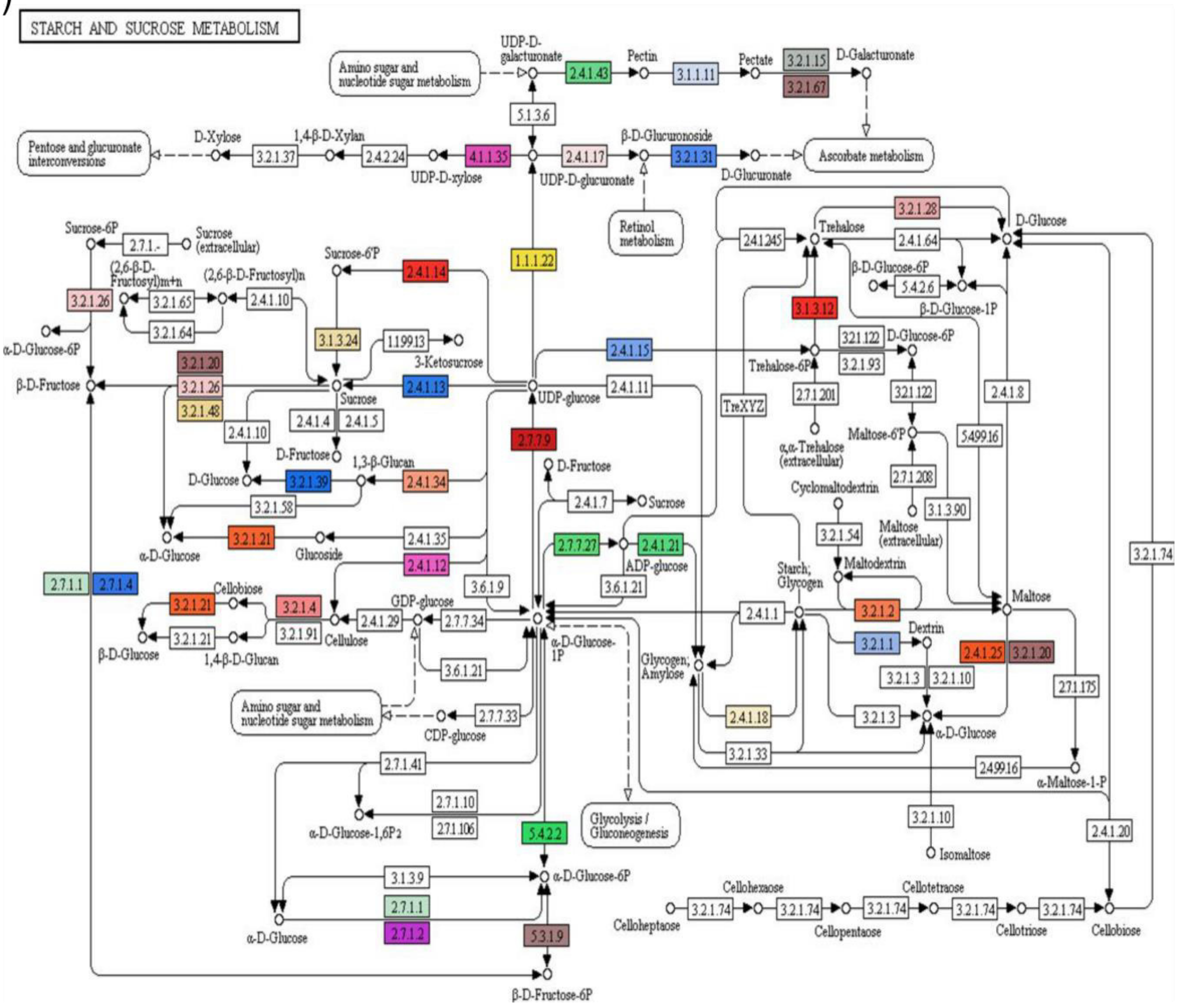

(b)

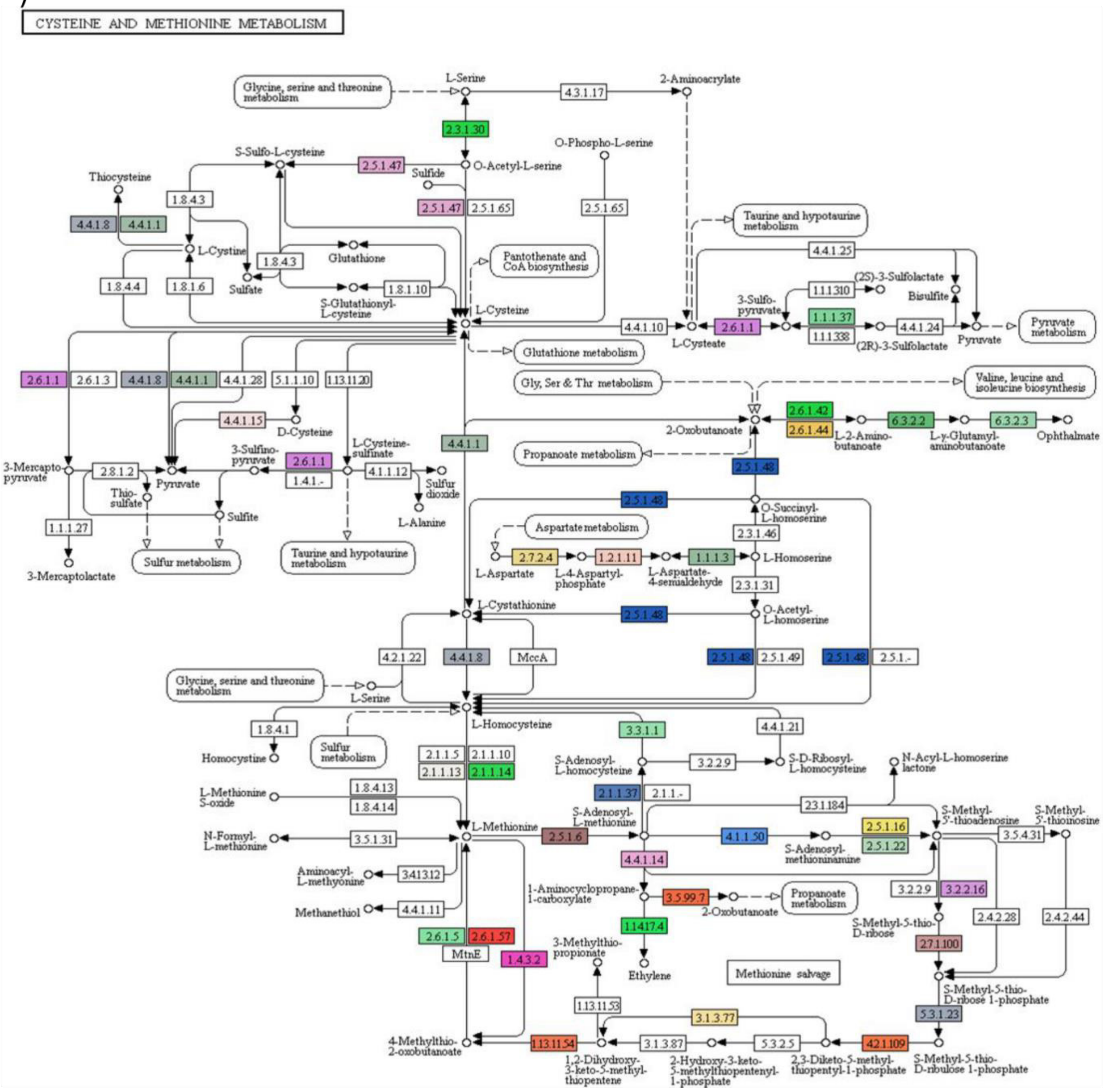
CYSTEINE AND METHIONINE METABOLISM
Glycine, serine and threonine metabolism
L-Serine
4.3.1.17
2-Aminoacrylate
2.3.1.30
S-Sulfo-L-cysteine
2.5.1.47
O-Acetyl-L-serine
O-Phospho-L-serine
Thiocysteine
Sulfide
1.8.4.3
2.5.1.47
2.5.1.65
2.5.1.65
4.4.1.8
4.4.1.1
Taurine and hypotaurine metabolism
L-Cystine
Glutathione
Pantothenate and CoA biosynthesis
4.4.1.25
1.8.4.3
Sulfate
S-Glutathionyl-L-cysteine
1.8.1.10
(2S)-3-Sulfolactate
1.1.1.310
Bisulfite
1.8.4.4
1.8.1.6
L-Cysteine
3-Sulfo-pyruvate
1.1.1.37
4.4.1.10
2.6.1.1
L-Cysteate
1.1.1.338
(2R)-3-Sulfolactate
4.4.1.24
Pyruvate
Pyruvate metabolism
Glutathione metabolism
2.6.1.1
2.6.1.3
4.4.1.8
4.4.1.1
4.4.1.28
5.1.1.10
1.13.11.20
Gly, Ser & Thr metabolism
Valine, leucine and isoleucine biosynthesis
4.4.1.15
D-Cysteine
4.4.1.1
2-Oxobutanoate
2.6.1.42
2.6.1.44
L-2-Amino-butanoate
6.3.2.2
L-γ-Glutamyl-aminobutanoate
6.3.2.3
Ophthalmate
3-Sulfino-pyruvate
L-Cysteine-sulfinate
Propanoate metabolism
3-Mercapto-pyruvate
2.8.1.2
2.6.1.1
1.4.1.-
4.1.1.12
Sulfur dioxide
2.5.1.48
Pyruvate
Thio-sulfate
Sulfite
L-Alanine
2.5.1.48
O-Succinyl-L-homoserine
1.1.1.27
Aspartate metabolism
2.3.1.46
3-Mercaptolactate
Sulfur metabolism
Taurine and hypotaurine metabolism
2.7.2.4
1.2.1.11
1.1.1.3
L-Homoserine
L-Aspartate
L-4-Aspartyl-phosphate
L-Aspartate-4-semialdehyde
2.3.1.31
L-Cystathionine
2.5.1.48
O-Acetyl-L-homoserine
4.2.1.22
4.4.1.8
MccA
2.5.1.48
2.5.1.49
2.5.1.48
2.5.1.-
Glycine, serine and threonine metabolism
L-Serine
L-Homocysteine
1.8.4.1
Sulfur metabolism
3.3.1.1
4.4.1.21
Homocystine
2.1.1.5
2.1.1.10
2.1.1.13
2.1.1.14
S-Adenosyl-L-homocysteine
3.2.2.9
S-D-Ribosyl-L-homocysteine
N-Acyl-L-homoserine lactone
L-Methionine S-oxide
1.8.4.13
1.8.4.14
2.1.1.37
2.1.1.-
2.3.1.184
S-Adenosyl-L-methionine
S-Methyl-5'-thioadenosine
S-Methyl-5'-thioinosine
N-Formyl-L-methionine
3.5.1.31
L-Methionine
2.5.1.6
4.1.1.50
S-Adenosyl-methioninamine
2.5.1.16
2.5.1.22
3.5.4.31
Aminoacyl-L-methyonine
3.4.13.12
4.4.1.14
3.2.2.9
3.2.2.16
1-Aminocyclopropane-1-carboxylate
3.5.99.7
2-Oxobutanoate
Propanoate metabolism
Methanethiol
4.4.1.11
S-Methyl-5-thio-D-ribose
2.4.2.28
2.4.2.44
1.14.17.4
2.6.1.5
2.6.1.57
3-Methylthio-propionate
2.7.1.100
MtnE
1.4.3.2
Ethylene
Methionine salvage
S-Methyl-5-thio-D-ribose 1-phosphate
1.13.11.53
5.3.1.23
3.1.3.77
4-Methylthio-2-oxobutanoate
1.13.11.54
3.1.3.87
5.3.2.5
4.2.1.109
1,2-Dihydroxy-3-keto-5-methyl-thiopentene
2-Hydroxy-3-keto-5-methylthiopentenyl-1-phosphate
2,3-Diketo-5-methyl-thiopentyl-1-phosphate
S-Methyl-5-thio-D-ribulose 1-phosphate

(c)

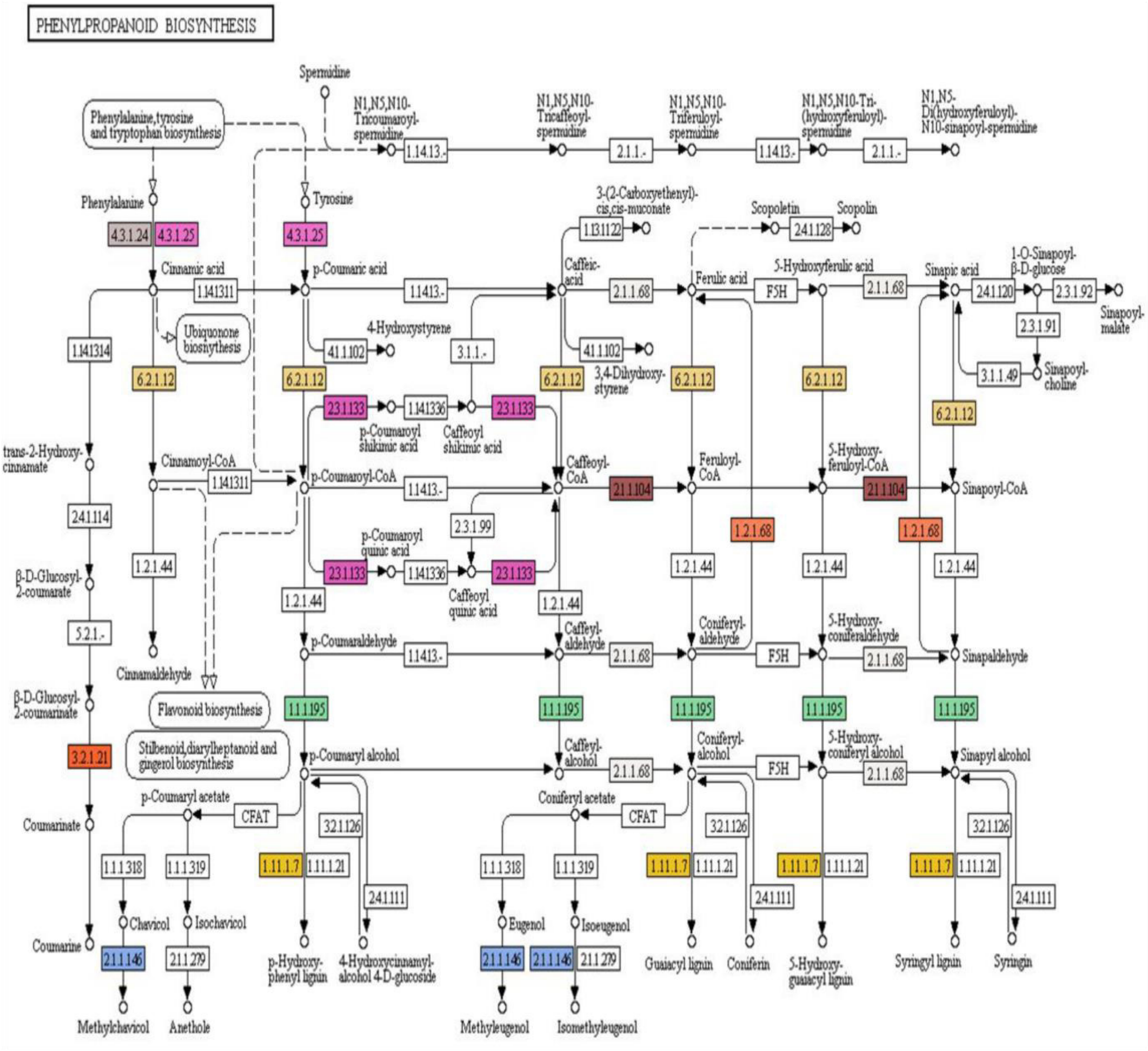
PHENYLPROPANOID BIOSYNTHESIS
Spermidine
Phenylalanine,tyrosine and tryptophan biosynthesis
N1,N5,N10-Tricoumaroyl-spermidine
N1,N5,N10-Tricaffeoyl-spermidine
N1,N5,N10-Triferuloyl-spermidine
N1,N5,N10-Tri-(hydroxyferuloyl)-spermidine
N1,N5-Di(hydroxyferuloyl)-N10-sinapoyl-spermidine
1.14.13.-
2.1.1.-
Phenylalanine
Tyrosine
4.3.1.24
4.3.1.25
Cinnamic acid
p-Coumaric acid
1.14.13.11
1.14.13.-
3-(2-Carboxyethenyl)-cis,cis-muconate
1.13.11.22
Scopoletin
Scopolin
2.4.1.128
Caffeic-acid
Ferulic acid
5-Hydroxyferulic acid
Sinapic acid
1-O-Sinapoyl-β-D-glucose
2.4.1.120
2.3.1.92
Sinapoyl-malate
2.3.1.91
3.1.1.49
Sinapoyl-choline
2.1.1.68
F5H
1.14.13.14
Ubiquonone biosynthesis
4-Hydroxystyrene
4.1.1.102
3.1.1.-
3,4-Dihydroxy-styrene
6.2.1.12
2.3.1.133
1.14.13.36
p-Coumaroyl shikimic acid
Caffeoyl shikimic acid
trans-2-Hydroxy-cinnamate
Cinnamoyl-CoA
p-Coumaroyl-CoA
Caffeoyl-CoA
Feruloyl-CoA
5-Hydroxy-feruloyl-CoA
Sinapoyl-CoA
2.1.1.104
1.2.1.68
2.4.1.114
2.3.1.99
p-Coumaroyl quinic acid
Caffeoyl quinic acid
1.2.1.44
β-D-Glucosyl-2-coumarate
5.2.1.-
Cinnamaldehyde
p-Coumaraldehyde
Caffeyl-aldehyde
Coniferyl-aldehyde
5-Hydroxy-coniferaldehyde
Sinapaldehyde
β-D-Glucosyl-2-coumarinate
Flavonoid biosynthesis
Stilbenoid,diarylheptanoid and gingerol biosynthesis
1.1.1.195
3.2.1.21
p-Coumaryl alcohol
Caffeyl-alcohol
Coniferyl-alcohol
5-Hydroxy-coniferyl alcohol
Sinapyl alcohol
p-Coumaryl acetate
Coniferyl acetate
CFAT
Coumarinate
3.2.1.126
1.1.1.318
1.1.1.319
1.11.1.7
1.11.1.21
2.4.1.111
Chavicol
Isochavicol
Eugenol
Isoeugenol
Coumarine
2.1.1.146
2.1.1.279
p-Hydroxy-phenyl lignin
4-Hydroxycinnamyl-alcohol 4-D-glucoside
Guaiacyl lignin
Coniferin
5-Hydroxy-guaiacyl lignin
Syringyl lignin
Syringin
Methylchavicol
Anethole
Methyleugenol
Isomethyleugenol

(d)

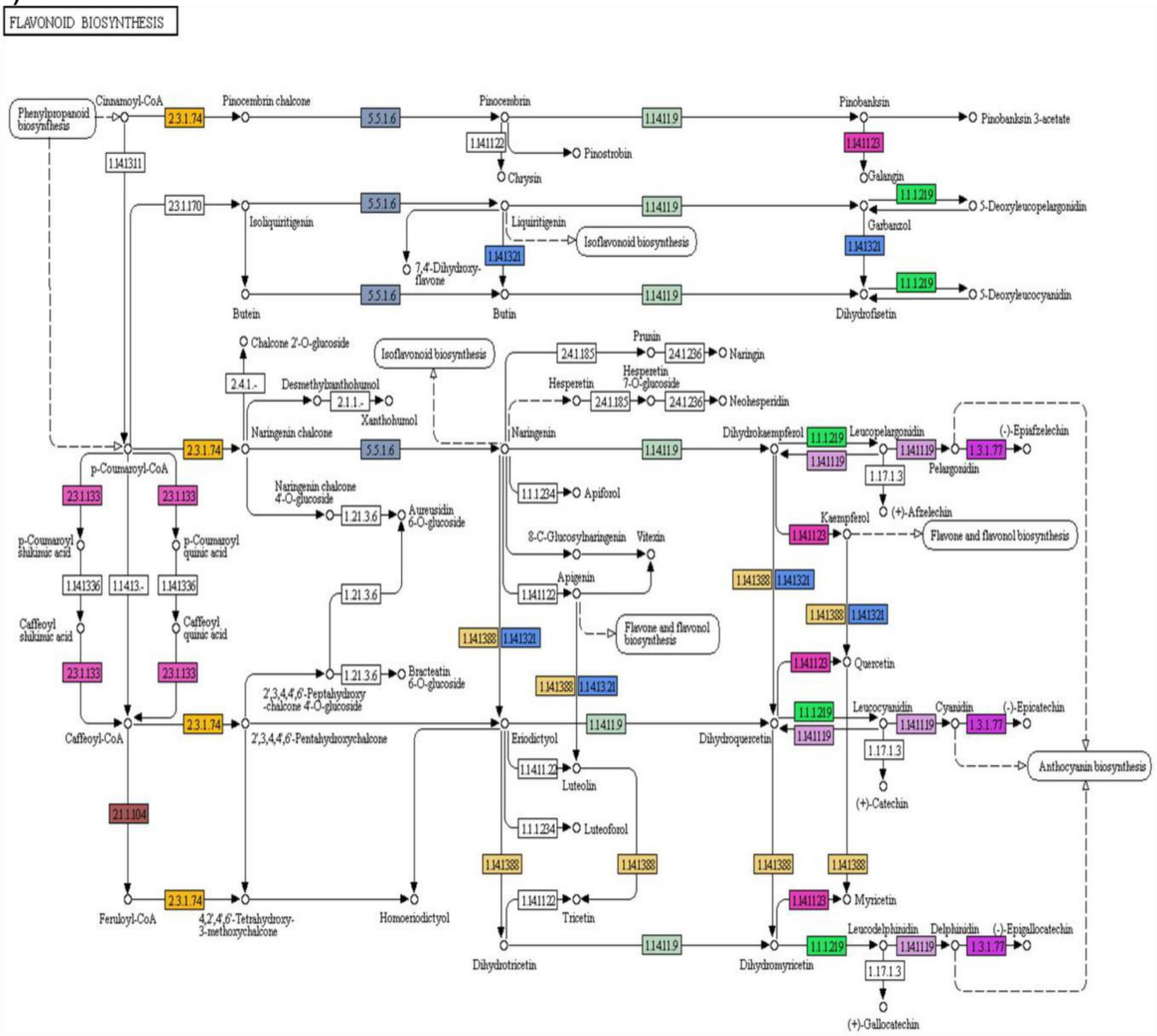
FLAVONOID BIOSYNTHESIS
Phenylpropanoid biosynthesis
Cinnamoyl-CoA
Pinocembrin chalcone
Pinocembrin
Pinobanksin
Pinobanksin 3-acetate
Pinostrobin
Chrysin
Galangin
Isoliquiritigenin
Liquiritigenin
Isoflavonoid biosynthesis
7,4'-Dihydroxy-flavone
5-Deoxyleucopelargonidin
Garbanzol
5-Deoxyleucocyanidin
Butein
Butin
Dihydrofisetin
Chalcone 2'-O-glucoside
Prunin
Naringin
Hesperetin
Hesperetin 7-O-glucoside
Neohesperidin
Desmethylxanthohumol
Xanthohumol
Naringenin chalcone
Naringenin
Dihydrokaempferol
Leucopelargonidin
(-)-Epiafzelechin
Pelargonidin
p-Coumaroyl-CoA
Naringenin chalcone 4'-O-glucoside
Aureusidin 6-O-glucoside
Apiforol
(+)-Afzelechin
Kaempferol
Flavone and flavonol biosynthesis
p-Coumaroyl shikimic acid
p-Coumaroyl quinic acid
8-C-Glucosylnaringenin
Vitexin
Apigenin
Caffeoyl shikimic acid
Caffeoyl quinic acid
Quercetin
Bracteatin 6-O-glucoside
2',3,4,4',6'-Peptahydroxy -chalcone 4'-O-glucoside
Leucocyanidin
Cyanidin
(-)-Epicatechin
Caffeoyl-CoA
2',3,4,4',6'-Pentahydroxychalcone
Eriodictyol
Dihydroquercetin
Anthocyanin biosynthesis
Luteolin
(+)-Catechin
Luteoforol
Myricetin
Feruloyl-CoA
4,2',4',6'-Tetrahydroxy-3-methoxychalcone
Homoeriodictyol
Tricetin
Leucodelphinidin
Delphinidin
(-)-Epigallocatechin
Dihydrotricetin
Dihydromyricetin
(+)-Gallocatechin
2.3.1.74
5.5.1.6
1.14.11.9
1.14.13.11
1.14.11.23
2.3.1.170
1.1.1.219
1.14.13.21
2.4.1.185
2.4.1.236
2.4.1.-
2.1.1.-
2.3.1.133
1.1.1.234
1.21.3.6
1.14.13.36
1.14.13.-
1.17.1.3
1.14.11.19
1.3.1.77
1.14.13.88
2.1.1.104

Supplementary Figure SF3: Phylogenetic analysis by neighbor-joining of proteins encoded by Enone oxidoreductase (gi|387135418) involved in the biosynthesis of furaneol from *Mangifera indica* (orange color) and other contigs representing quinone oxidoreductases from the present study (blue color) and other characterized oxidoreductases (black color) from *Nicotiana tabacum*_2-alkenal_reductase_(gi|75206691), *Fragaria vesca*_Enone_oxidoreductase (gi|613785129), *Solanum lycopersicum*_2-methylene-furan-3-one_reductase (gi|823630988), *Fragaria* × *Ananassa*_ Enone_Oxidoreductase (gi|480312155) and *Fragaria* × *Ananassa*_ quinone_oxidoreductase (gi|29468088)

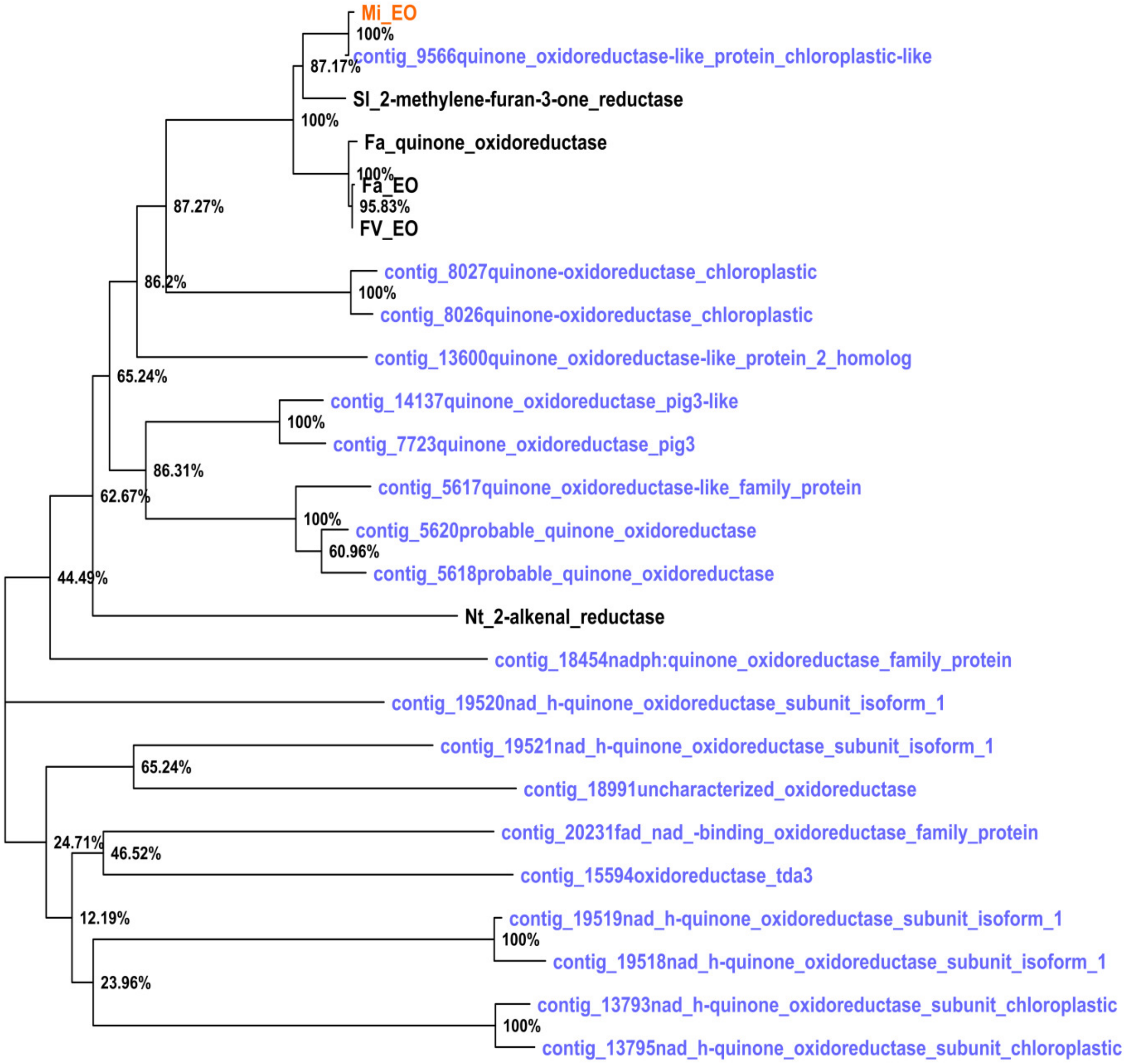

Supplementary Figure SF4: Phylogenetic analysis by neighbor-joining of proteins encoded by *O-methyltransferase* (KP993176) involved in the biosynthesis of mesifuran from *Mangifera indica* (orange color) along with the characterized *O-methyltransferase* (black color) from *Fragaria x ananassa* (AAF28353) and other contigs representing *O-methyltransferase* from the present study (blue color).

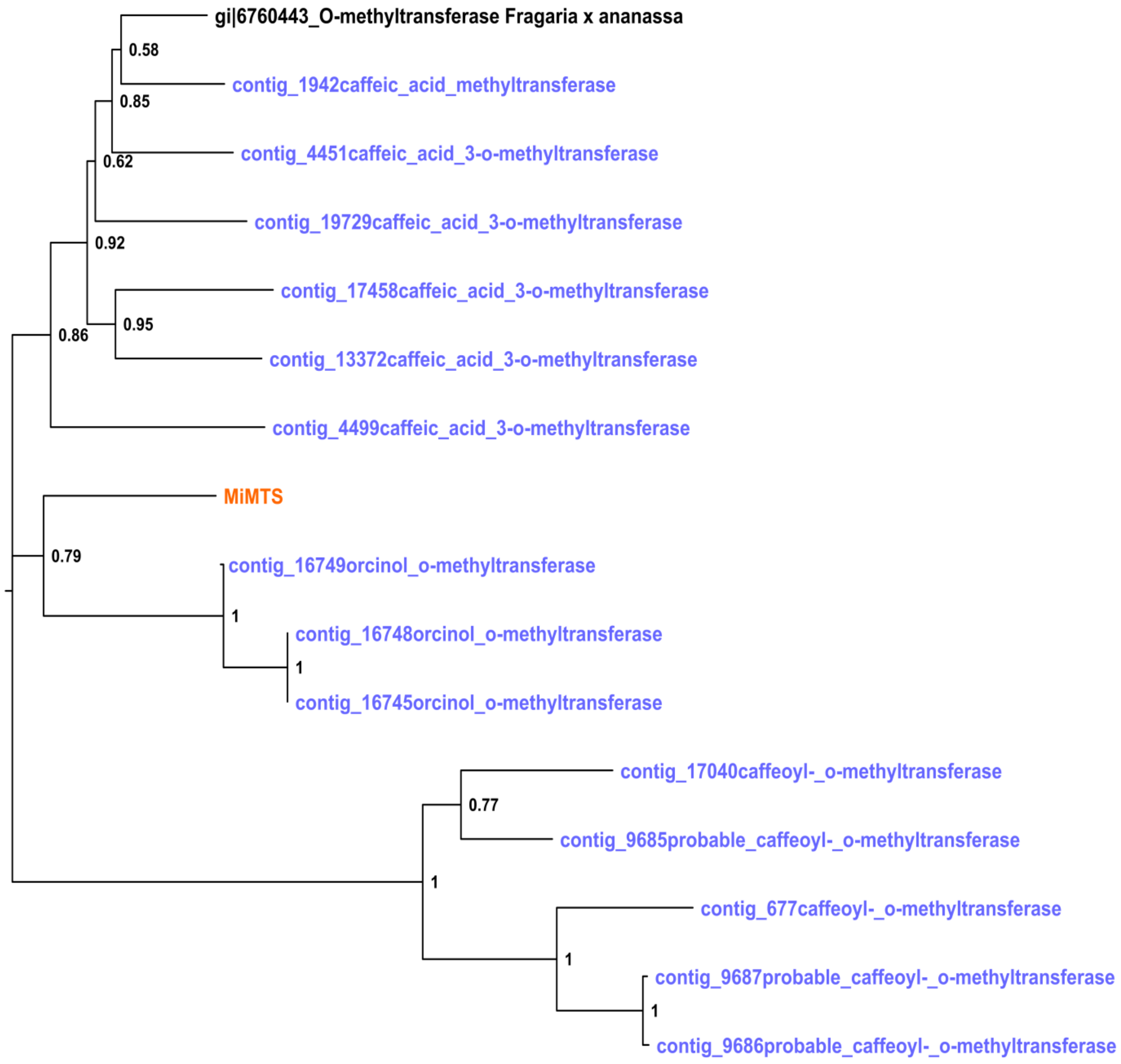

Supplementary Figure SF5: Phylogenetic analysis by neighbor-joining of proteins encoded by *9-lipoxygenase* (KX090178) involved in the biosynthesis of aroma volatiles from *Mangifera indica* (orange color) and other contigs representing *lipoxygenases* from present study (blue color) along with the other plant *9 and 13 lipoxygenases* (black color) from *Vitis vinifera*_9LOX (XP_010659859), *Malus domestica*_9LOX (NP_001281030), *Prunus mume*_9LOX (XP_008246456), *Glycine max*_9LOX (XP_003521704), *Solanum tuberosum*_9LOX (NP_001274916), *Nicotiana tabacum*_9LOX (XP_016433823), *Dimocarpus longan*_9LOX (ANF89411), *Corylus avellana*_9LOX (CAD10740), *Litchi chinensis*_9LOX (AEQ30071), *Populus euphratica*_9LOX (XP_011023610), *Eucalyptus grandis*_9LOX (XP_010025195), Jatropha curcas_9LOX (XP_012089053), *Ricinus communis*_9LOX (XP_002512386), *Solanum lycopersicum*_9LOX (XP_004244890), *Sesamum indicum*_9LOX (XP_011087404), *Nelumbo nucifera*_9LOX (XP_010256003), *Malus domestica*_13LOX (NP_001280985), *Prunus mume*_13LOX (XP_008228181), *Medicago truncatula*_13LOX (XP_003627308), *Solanum tuberosum*_13LOX (NP_001275115), *Glycine soja*_13LOX (KHN39622), *Fragaria vesca*_13LOX (XP_004303702), *Morus notabilis*_13LOX (XP_010086794), *Eucalyptus grandis*_13LOX (XP_010033729), *Populus euphratica*_13LOX (XP_011035732), *Nelumbo nucifera*_13LOX (XP_010273845), *Capsicum annuum*_13LOX (NP_001311748), *Nicotiana tabacum*_13LOX (XP_016495606), *Citrus sinensis*_13LOX (XP_006465905), *Solanum lycopersicum*_13LOX (AAB65767) and *Glycine max*_13LOX (XP_014624448).

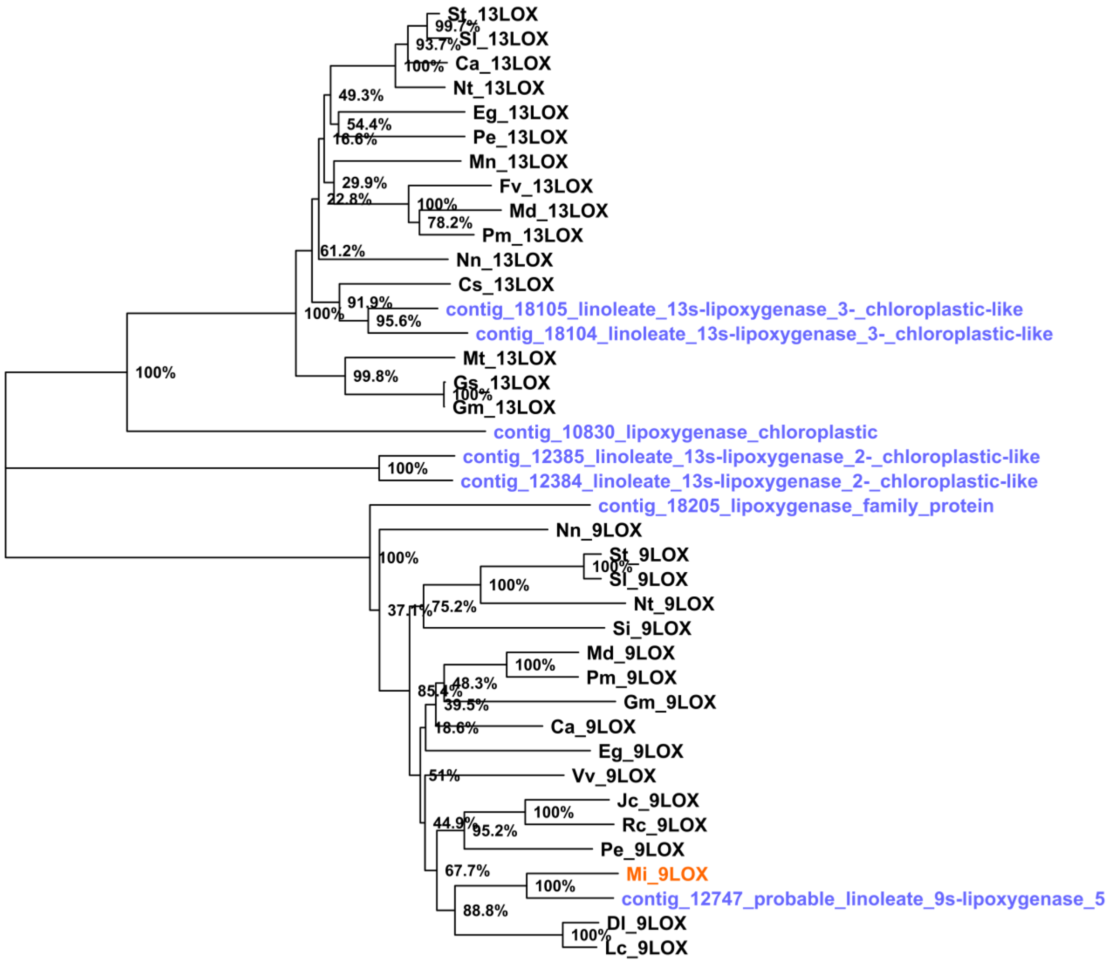

Supplementary Figure SF6: Phylogenetic analysis by neighbor-joining of proteins encoded by *epoxide hydrolase 2* (KX090179) involved in the biosynthesis of aroma volatile lactones from *Mangifera indica* (orange color) and other contigs representing *epoxide hydrolases* from the present study (blue color) along with the other plant *epoxide hydrolases* (black color) from *Nicotiana benthamiana*_EH1.2 (ACE82566), *Nicotiana benthamiana*_EH1.1 (ACE82565), *Phaseolus vulgaris*_EH (AKJ75509), *Glycine max*_EH (CAA55294), *Medicago truncatula*_EH (XP_003626202), *Glycine soja*_EH (KHN43314), *Vigna radiata*_EH (AIJ27456), *Brassica napus*_EH (NP_001302895), *Citrus sinensis*_EH2 (XP_006489040), *Prunus mume*_EH2 (XP_016647751), *Cicer arietinum*_EH2 (XP_004508197), Fragaria vesca_EH2 (XP_004290776), *Cajanus cajan*_EH2 (KYP58120), *Cucumis sativus*_EH2 (XP_004134492), *Solanum lycopersicum*_EH2 (XP_004252913) and *Morus notabilis*_EH2 (XP_010105136).

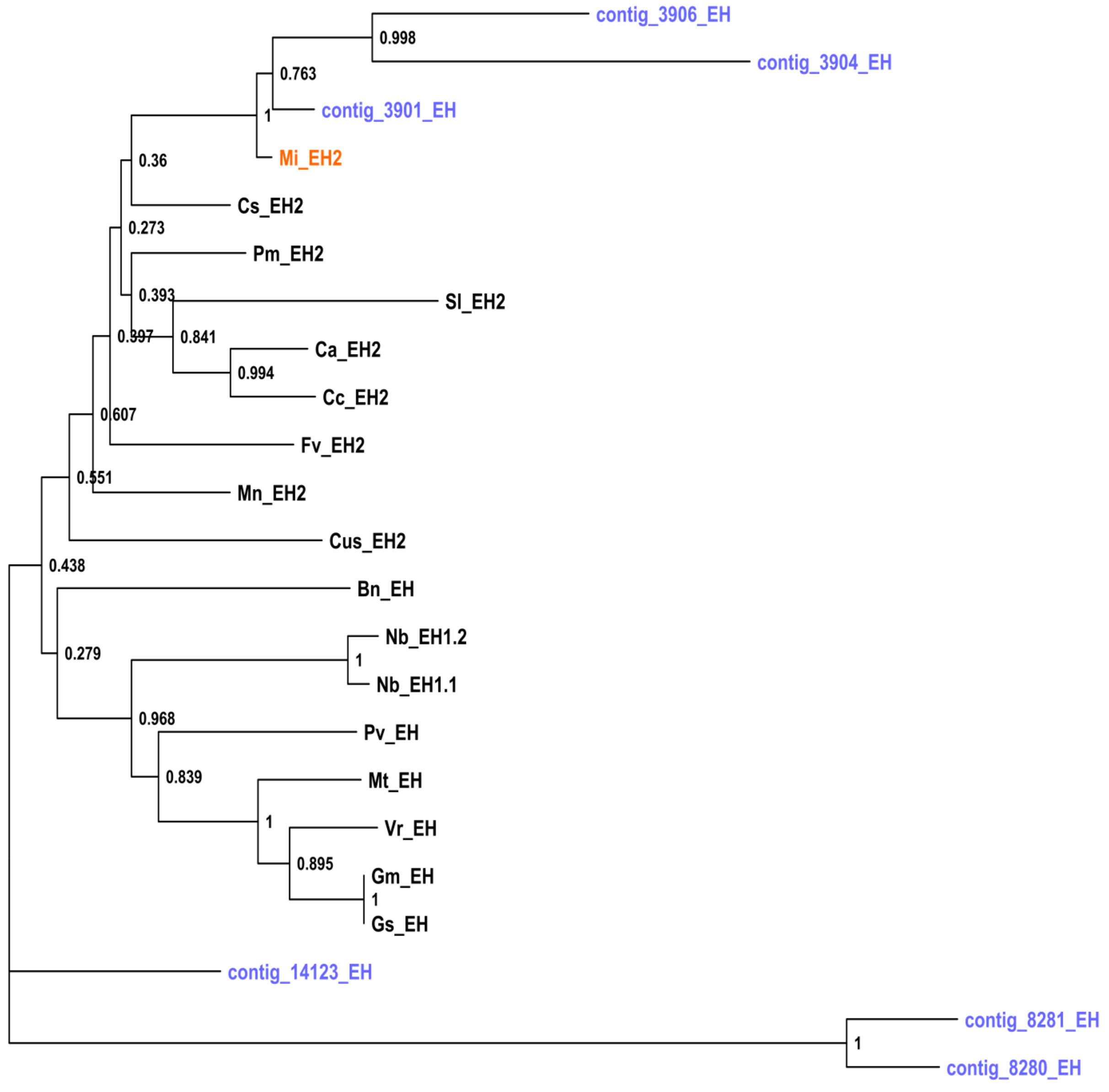

Supplementary Figure SF7: Heatmap representing differential expression of various contigs coding for genes involved in aroma biosynthesis (TPS: terpene synthase, EH: epoxide hydrolase, LOX: lipoxygenase, O-MTS: O-methyltransferase and EO: enone oxidoreductase) through various stages.

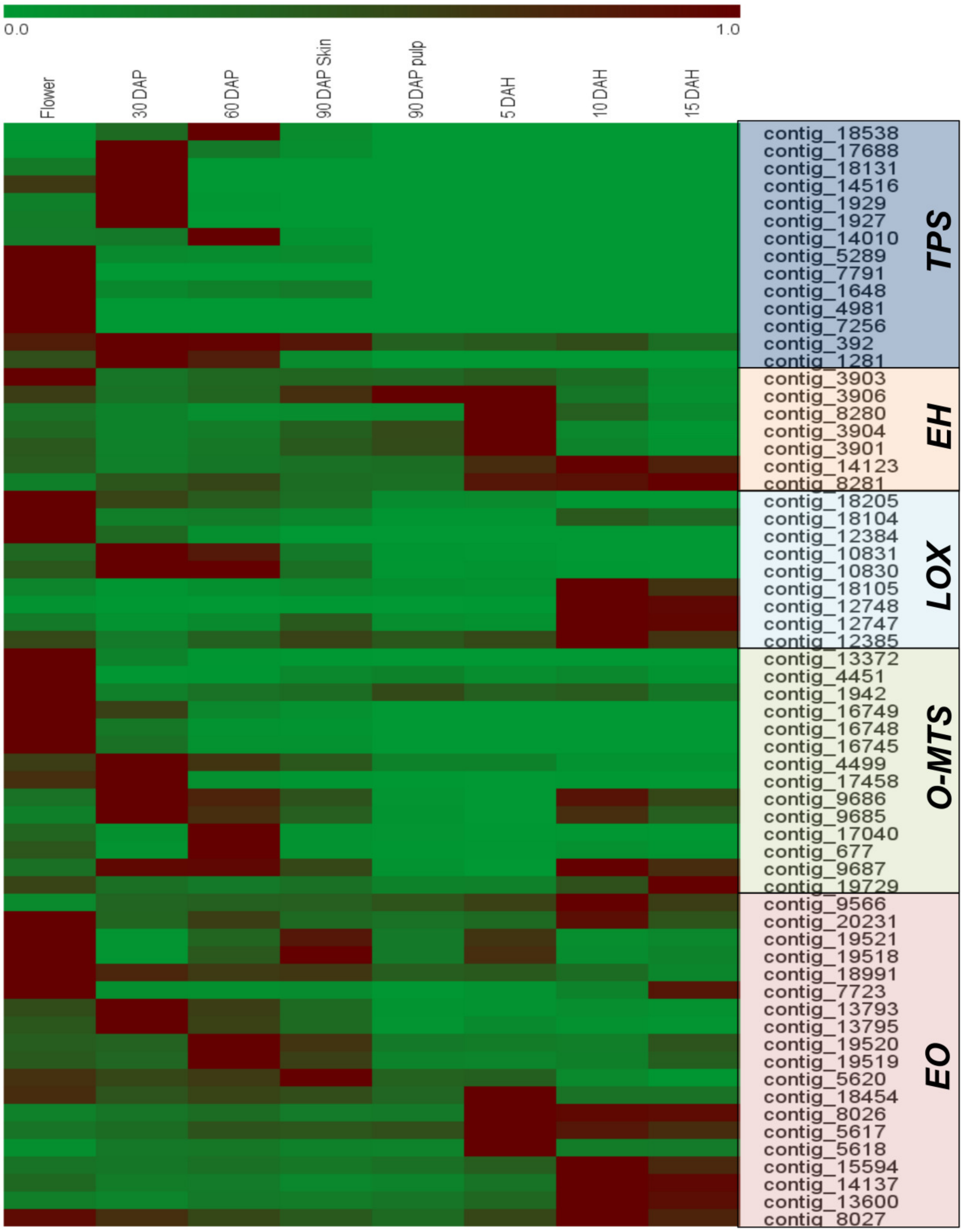

Supplementary Figure SF8: Heatmap representing differential expression of various contigs coding for 20 different inhibitors. Description for group 1-20 as follows, 1: alpha-amylase inhibitor, 2: androgen induced inhibitor of proliferation pds5, 3: apoptosis inhibitor, 4: bax inhibitor, 5: bifunctional inhibitor of lipid-transfer protein seed storage, 6: cell wall and vascular inhibitor of beta-fructosidase, 7: kinase inhibitor, 8: cysteine proteinase inhibitor , 9: trypsin inhibitor, 10: guanosine nucleotide diphosphate dissociation inhibitor, 11: macrophage migration inhibitory factor, 12: nf-kappa-b inhibitor, 13: pectinesterase inhibitor, 14: polygalacturonase inhibiting protein, 15: phosphoprotein phosphatase inhibitors, 16: plasminogen activator inhibitor 1 rna-binding, 17: proteasome inhibitor, 18: protein reversion to ethylene sensitivity, 19: protein transport inhibitor and 20: rho gdp-dissociation inhibitor.

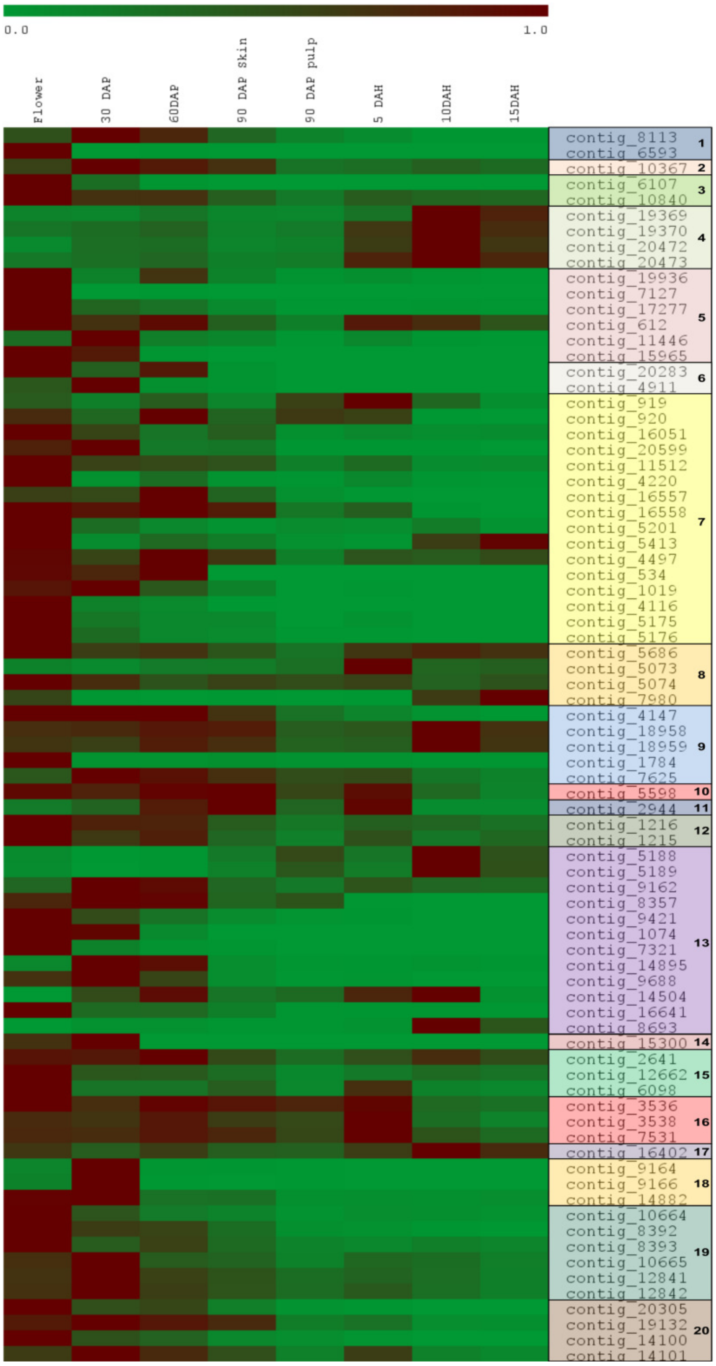

Supplementary Figure SF9: Terpene biosynthesis pathway from Alphonso mango

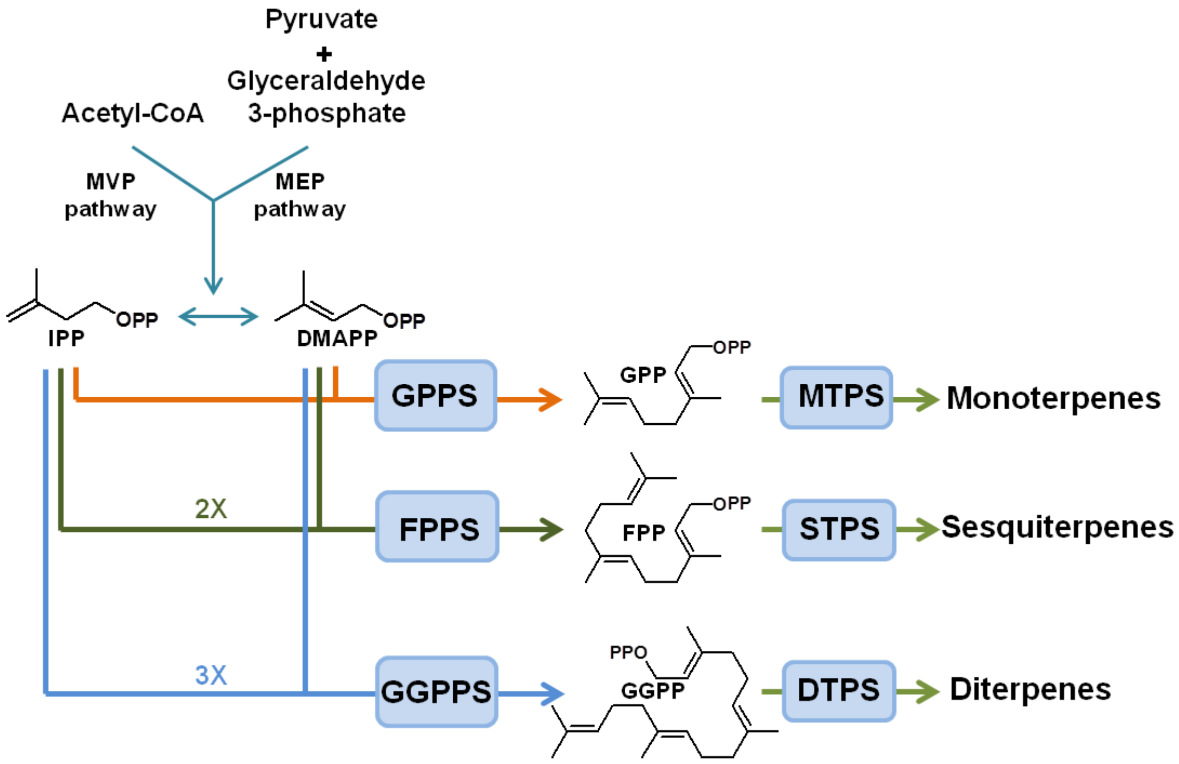

Supplement: Supplementary file 1 — Supplementary information [file 41598_2017_8499_MOESM1_ESM.pdf]
